# Histone Deacetylases Control Neurogenesis in Embryonic Brain by Inhibition of BMP2/4 Signaling

**DOI:** 10.1371/journal.pone.0002668

**Published:** 2008-07-16

**Authors:** Maya Shakèd, Kathrin Weissmüller, Hanno Svoboda, Peter Hortschansky, Norikazu Nishino, Stefan Wölfl, Kerry L. Tucker

**Affiliations:** 1 Interdisciplinary Center for Neurosciences, University of Heidelberg, Heidelberg, Germany; 2 Leibniz Institute for Natural Product Research and Infection Biology, Hans Knöll Institute, Jena, Germany; 3 Graduate School of Life Science and Systems Engineering, Kyushu Institute of Technology, Kitakyushu, Japan; 4 Institute of Pharmacy and Molecular Biotechnology, University of Heidelberg, Heidelberg, Germany; University of Sydney, Australia

## Abstract

**Background:**

Histone-modifying enzymes are essential for a wide variety of cellular processes dependent upon changes in gene expression. Histone deacetylases (HDACs) lead to the compaction of chromatin and subsequent silencing of gene transcription, and they have recently been implicated in a diversity of functions and dysfunctions in the postnatal and adult brain including ocular dominance plasticity, memory consolidation, drug addiction, and depression. Here we investigate the role of HDACs in the generation of neurons and astrocytes in the embryonic brain.

**Principal Findings:**

As a variety of HDACs are expressed in differentiating neural progenitor cells, we have taken a pharmacological approach to inhibit multiple family members. Inhibition of class I and II HDACs in developing mouse embryos with trichostatin A resulted in a dramatic reduction in neurogenesis in the ganglionic eminences and a modest increase in neurogenesis in the cortex. An identical effect was observed upon pharmacological inhibition of HDACs in *in vitro*-differentiating neural precursors derived from the same brain regions. A reduction in neurogenesis in ganglionic eminence-derived neural precursors was accompanied by an increase in the production of immature astrocytes. We show that HDACs control neurogenesis by inhibition of the bone morphogenetic protein BMP2/4 signaling pathway in radial glial cells. HDACs function at the transcriptional level by inhibiting and promoting, respectively, the expression of *Bmp*2 and *Smad7*, an intracellular inhibitor of BMP signaling. Inhibition of the BMP2/4 signaling pathway restored normal levels of neurogenesis and astrogliogenesis to both ganglionic eminence- and cortex-derived cultures in which HDACs were inhibited.

**Conclusions:**

Our results demonstrate a transcriptionally-based regulation of BMP2/4 signaling by HDACs both *in vivo* and *in vitro* that is critical for neurogenesis in the ganglionic eminences and that modulates cortical neurogenesis. The results also suggest that HDACs may regulate the developmental switch from neurogenesis to astrogliogenesis that occurs in late gestation.

## Introduction

Neurons are the predominant terminally-differentiated cell type produced in the brain during prenatal development in vertebrates [1]. In the developing cortex, glutamatergic projection neurons are generated that then migrate radially outward to assume their proper position in one of the six layers of the postnatal neocortex. Cortical neurons arise from proliferating radial glia cells [2], [3], either directly generated in an asymmetrical fashion at the pseudostratified ventricular zone or deriving from a symmetrically-dividing basal progenitor, also generated by radial glia, in the subventricular zone [4]–[6]. In the lateral and medial ganglionic eminences (GE) of the embryonic striatum and pallidum, respectively, GABAergic interneurons are generated to a large extent in the subventricular zone, and many of these neurons proceed to leave the GE, migrating tangentially and populating the cortex (reviewed in [7], [8]). Radial glia also contribute to neurogenesis in the GE [2]. The progenitor populations in the cortex and GE express a distinctly different palette of transcription factors to direct regional-specific neurogenesis. For example, Pax6 and Ngn2 expression in the dorsal telencephalon leads to a cortical differentiation program including the expression of the proneural transcription factors Math2/3, NeuroD1/2, and Tbr1/2, whereas Mash1 and Nkx2.1 expression in the ventral telencephalon leads to a striatal / pallidal differentiation program marked by the expression there of the homeobox genes Dlx1/2, Dlx5/6, and Gsh1/2, respectively [7].

In mammals, astrocytes and oligodendrocytes are first born late in gestation and continue to be produced well after birth [1]. The developmental transition from a primarily neurogenic to an astrogliogenic program is only partially understood. The emergence of astrogliogenic precursors is actively suppressed by the basic helix-loop-helix transcription factors Ngn2 and Mash1 [9], [10]. Two key signal transduction pathways are involved in the promotion of astrogliogenesis. Leukemia inhibitory factor (LIF) and ciliary neurotrophic factor (CNTF) activate the JAK-STAT pathway through their common receptor gp130 [11], [12], while the bone morphogenetic proteins 2 and 4 (BMP2/4), binding to their co-receptors BMPR1A/B and BMPR2, activate the Smad1/5/8 pathway (reviewed in [13]) Both of these pathways converge *in vitro* at the promoter for glial fibrillary acidic protein (GFAP), a marker of newborn, maturing astrocytes [12]. BMPs are a family of secreted growth factors belonging to the TGFβ superfamily. BMP2 and BMP4 promote the generation of astrocytes both *in vitro* and *in vivo* [14], [15], partially by the induction of the transcription factors Hes5, Id1, and Id3, which antagonize the pro-neurogenic activity of Ngn1 [16]. However, the importance of BMPs for neurogenesis in the brain is still unclear. Multiple BMPs [17] and their receptors [18], [19] are expressed in neurogenic regions of the developing brain, starting as early as 10.5 d.p.c. in the telencephalon [18] and extending throughout the major period of neurogenesis in the developing cortex and the GE [19]. Initial experiments indicated that ectopically applied BMP2 and -4 promote neurogenesis in the cortex [20], [21], but BMP2 and -4 were shown to inhibit neurogenesis in the embryonic and adult striatum [14], [22] and interneuron development in the embryonic cortex [23]. Analyses using mice deficient in the BMP receptor *Bmpr1a* [24] suggested a minimal role in the regulation of cortical neurogenesis. However, recent reports clearly documented the loss of DI1 and DI2 interneurons in the spinal cord [25] and of cerebellar granule neurons in the cerebellum [26] of mice lacking both *Bmpr1a* and *Bmpr1b*. Ablation of *Bmpr1a* and *Bmpr1b* in the telencephalon resulted in holoprosencephaly and embryonic death by 11.5 d.p.c. [27], thereby precluding analysis of the importance of BMP2 and 4 for the major periods of neurogenesis and astrogliogenesis.

The modification of chromatin is a central aspect of the regulation of gene transcription in eukaryotic cells. The covalent modification of core histones by methylation, phosphorylation, and acetylation forms a combinatorial “code” that governs the transcriptional activity of DNA sequences wound around histone octamers [28]. Acetylation of the ε-amino groups of lysine residues in the amino-termini of core histones by histone acetyltransferases (HATs) neutralizes the positive charges that normally stabilize the formation of the compacted 30-nm fiber and inter-fiber interactions [29]. Thus, histone acetylation leads to a decondensed nucleosomic structure that allows access of transcription factors to the DNA. Conversely, transcriptional inactivity is associated with the removal of acetyl groups by histone deacetylases (HDACs), a large group of enzymes classified into three gene families [30]. Class I HDACs (HDAC1, -2, -3, and -8) are orthologs of the yeast RPD3 protein and are almost exclusively localized in the nucleus. They have been shown to co-localize in complexes such as Sin3, NuRD, and Co-REST that potentiate HDAC activity and contain transcriptional co-repressors [31], [32]. HDAC1 also associates with the methylCpG-binding protein MeCP2 [33], providing a functional link between DNA methylation and histone modification. Class II HDACs (HDAC4, -5, -6, -7, -9, and -10) are homologous to the yeast HDA1 protein, are double the size of the class I HDACs, and can shuttle between the nucleus and the cytoplasm [34]. Class II HDACs interact with the transcriptional co-repressors N-CoR and SMRT [35] and the MEF2 family of transcription factors [36]. Class I and II HDACs share almost identical catalytic domains, whereas the NAD^+^-dependent class III HDACs form an evolutionarily distinct family homologous to yeast Sir2 proteins [37].

The importance of histone acetylation for dynamic regulation of gene expression during development has been intensively investigated for skeletal and cardiac myogenesis. The class II HDACs HDAC4, -5, and -7 associate with the myogenic transcription factor MEF2 and thereby inhibit its activity in the nucleus [36]. Myogenic signals lead to the phosphorylation of these HDACs by CaM kinase [38] and, after binding of 14-3-3 proteins, export from the nucleus [39], [40]. The HAT p300 can then associate with MEF2, and also MyoD [41], to promote the activation of muscle-specific genes. Evidence is now emerging that HDACs are also important for the development of the nervous system as well. HDACs have been shown to promote the genesis [42] and maturation [43], [44] of oligodendrocytes in the rat [43]–[45] and in zebrafish [42]. Two reports have shown that the class I *hdac1* promotes the generation of neurons in the retina [46] and the spinal cord [47] of the zebrafish. Evidence for the role of HDACs in the control of neurogenesis and astrogliogenesis in the mammalian brain, however, is lacking. Although ablation of *Hdac4* can occasionally lead to exencephaly [48], other gene knockouts in the mouse have resulted in either early embryonic lethality (*Hdac1* [49]), or in no reported change in brain development (*Hdac5* [50], *Hdac9* [51], *Hdac7* [52], and *Hdac2* [53]). This may be explained by the large number of HDACs that are expressed in the developing mammalian brain [44], which could allow for functional redundancy.

We investigated the role of HDACs in the generation of neurons and astrocytes in developing brain. As a large number of HDACs are expressed in differentiating neural stem cells and progenitors, we employed a pharmacological approach to inhibit all members of the class I and II families. Treatment of mouse embryos with the highly-specific HDAC inhibitor trichostatin A caused a dramatic reduction in neurogenesis in the GE and a modest increase in the cortex. An identical effect was observed in *in vitro*-differentiated neural precursor cultures prepared from embryonic GE and cortex [54]. We demonstrate that HDACs control the transcription of *Bmp2* itself and *Smad7*, an inhibitor of BMP signaling [55], whose expression is inhibited and promoted by HDACs, respectively. HDACs promote neurogenesis in the GE by downregulation of BMP2/4 signaling, leading to the favored production of neurons over astrocytes, whereas HDAC-mediated inhibition of BMP2/4 signaling in the cortex leads to an opposite outcome.

## Results

### HDAC function is necessary for neurogenesis in the ganglionic eminences *in vitro*

**Figure 1:**
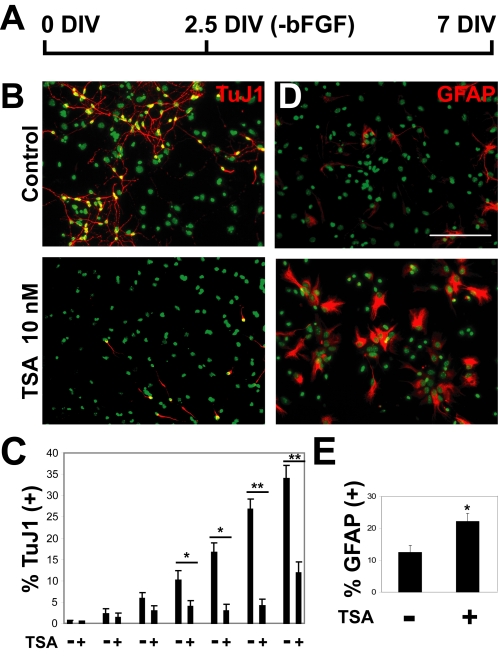
Inhibition of HDACs by TSA treatment blocks neurogenesis in differentiating neural progenitor cultures derived from embryonic GE. (A) Neurosphere cultures derived from 15.5 d.p.c. mouse GE were dissociated, cultured on polyornithine, and the mitogen bFGF was removed from the cultures at 2.5 days *in vitro* (DIV). Neurogenesis was evaluated at 7 DIV. (B) 7 DIV cultures were stained with the TuJ1 antibody (red) to detect β-tubulin III expression, marking newborn neurons, and DAPI (green) to stain cell nuclei. (C) Time course of suppression of neurogenesis by TSA. Cultures were stained at each day of the 1-week differentiation period and the percentage of neurons produced was calculated using a DAPI stain to evaluate total cell number. Cultures were untreated (−) or treated with 10 nM TSA (+). (D) The cultures described in (B) were also stained for astrocytes with an antibody recognizing GFAP (red) and DAPI (green) to stain cell nuclei. (B, D) Scale bar = 100 µm. (E) The percentage of cells detected with the GFAP antibody is indicated. (C, E) Mean values +/− SEM (n = 5). * = p<0.05, ** = p<0.01, Mann-Whitney U test.

In order to perform a molecular biological and biochemical analysis of the effects of HDAC inhibition upon neurogenesis in the developing embryo, an *in vitro*-based approach was at first selected. To generate a uniform population of neural precursors directly from embryonic brain, we turned to the well-established technique of neurosphere cultures [54]. We performed *in vitro* differentiation upon precursor cells derived from embryonic lateral and medial ganglionic eminences (GE). Neurosphere cultures were prepared from the GE from mice of the inbred C57BL/6J background at 15.5 d.p.c., cultured for seven days as floating cell aggregates, and then differentiated for seven days in monolayer culture on polyornithine-coated cover slips, according to standard methods, in a medium that supports the differentiation of both neurons and astrocytes [56]. To promote differentiation, the mitogen basic FGF (bFGF) was withdrawn after 2.5 days *in vitro* (DIV; Fig. 1A). We first utilized Western blots to examine protein expression of class I (HDAC1, -2, -3) and class II HDACs (HDAC4, -5, -6, -7) in these cultures. All examined HDACs were expressed throughout the period of differentiation, with a general decrease in expression levels over the course of time, with the exception of HDAC4 and 6, which maintained and increased, respectively, their level of protein expression (Fig. S1A). We can clearly attribute the expression of HDACs to neural precursors at early stages of the cultures, as they comprise more than 90% of the cell population after plating the neurospheres out for monolayer culture, as judged by positive staining for nestin and cellular morphology (Fig. S1B, C).

As neural precursors express a wide variety of class I and II HDACs, we decided to adopt a pharmacological approach to inhibit both families. We employed trichostatin A (TSA), a highly specific *Streptomyces*-derived hydroxamic acid that interacts at nanomolar concentration with the catalytic site of class I and II HDACs, acting as a potent, reversible, competitive inhibitor [57]. 10 nM TSA was added to the medium upon plating out of dissociated neurospheres and again at 2.5 DIV, when bFGF was withdrawn (Fig. 1A). After an additional 4.5 days (7 DIV) cells were fixed and stained with the TuJ1 antibody to detect β-tubulin III expression, a marker for newborn neurons, evaluating cell number by counting DAPI-stained nuclei. The percentage of neurons dramatically decreased from 35% in untreated cultures to 12% upon 10 nM TSA treatment (Fig. 1B, C),. A statistically significant decrease could be observed from the fourth day of differentiation (Fig. 1C and Table 1). The percentage of neurons formed did not increase if cultures were differentiated an additional 3 days in the absence of TSA (Control: 31.2±1.5%; TSA: 7.9±0.6%, n = 2).

**Table 1:** Time-course of suppression of neurogenesis by TSA, showing percentage of cells that become neurons, as seen in Fig. 1C.

Days *in vitro*	1 DIV	2 DIV	3 DIV	4 DIV	5 DIV	6 DIV	7 DIV
**Control**	0.6±0.8	2.3±1.1	5.9±1.3	10.2±2.1	16.7±2.2	26.8±2.4	34.0±3.1
**TSA 10 nM**	0.4±0.6	1.4±1.0	3.0±1.1	4.0±1.4 *	3.0±1.4*	4.1±1.6**	11.8±2.6**
**VPA 0.3 mM**	0.6±0.7	2.7±1.2	8.3±1. 7	15.1±2.0	13.7±2.2	25.1±2.1	34.0±2.6

Mean values +/− SEM (n = 3–6). ^*^ = p<0.05, ^**^ = p<0.01, ^***^ = p<0.001, Mann-Whitney U test.

We employed a wide range of HDAC inhibitors, including the hydroxamic acids SAHA and SBHA and the cyclic tetrapeptide apicidin. All of these inhibitors were able to inhibit neurogenesis, in all cases completely eliminating neurogenesis at nanomolar or micromolar concentrations (Fig. S2A). Interestingly, valproic acid (VPA, 2-propylpentanoic acid), a short-chained fatty acid that preferentially inhibits class I HDACs [58], [59] and has long been used as an anti-epileptic drug, did not show a reduction in neurogenesis (Table 1), even when used at 1.0 mM, a concentration well above the IC_50_ range for inhibition of Class I HDACs [58].

To address the possibility that HDAC inhibition through TSA may inhibit the expression of β-tubulin III in neurons, we also prepared neurosphere cultures from transgenic mice (the *tauGFP* line) with an insertion of EGFP at the tau locus, which is expressed specifically in newborn neurons in a time frame similar to induction of β-tubulin III [60]. Using antibodies to GFP to identify neurons, a similar reduction in neurogenesis was observed upon treatment with 10 nM TSA (Control: 32.5±2.4%; TSA: 10.8±4.1%, n = 4). Finally, we prepared neurosphere cultures from wild-type mice of the CBA/J background and examined neurogenesis after treatment with TSA in order to test the specificity of this effect for a given genetic background. Again, 10 nM TSA application reduced neurogenesis, from 33.8% in control conditions to 10.8% upon application of TSA. The inhibition of neuron production by TSA showed a concentration-dependent effect, with increasing concentrations resulting in fewer neurons, dropping down to only 4.3% at 50 nM (Table 2).

**Table 2:** Dose-dependent suppression of neurogenesis by TSA.

Treatment	Control	TSA 2 nM	TSA 10 nM	TSA 50 nM
**% neurons**	30.8±1.9	22.6±2.3	9.7±1.2 **	4.3±1.5 ***

Treatment of neurosphere cultures with the indicated TSA concentrations for 1 week during *in vitro* differentiation revealed a dose-dependent effect upon the production of neurons. Mean values +/− SEM (n = 3). p values are indicated as in Table 1.

Neural precursors derived from neurospheres can differentiate into neurons, oligodendrocytes and astrocytes, or they can remain as proliferating neural precursors [61]. To determine if the HDAC inhibitors had an effect on the production of the latter three cell types, we stained cells after a week of differentiation with antibodies against glial fibrillary acidic protein (GFAP), which recognizes astrocytes; nestin, a marker for neural precursors; and O4, a marker for newborn oligodendrocytes. Astrocytic differentiation almost doubled in TSA-treated cells, changing from 11.9±2.0% in untreated cells to 22.3±2.4% in 10 nM TSA-treated cells (Fig. 1D, E; n = 5). In addition, three other HDAC inhibitors, the hydroxamic acids SAHA and SBHA and the cyclic tetrapeptide apicidin, all increased astrogliogenesis in a dose-dependent fashion (Fig. S2B). During the first 3 days of differentiation over 80% of the cells expressed nestin in both TSA-treated and untreated cells (Fig. S1B, C, D). This expression was reduced over the course of a week to 19.1±2.1% nestin (+) in the control cells (Fig. S3A, C), reflecting the production of differentiated neurons, astrocytes, and oligodendrocytes. In contrast, 10 nM TSA treatment resulted in 25.9±3.3% of the cells remaining nestin positive (Fig. S3A, C; n = 3). Finally, after treatment with 10 nM TSA, the percentage of oligodendrocytes decreased from 7.3±1.6% to 1.7±0.5% (Fig. S3B, D; n = 3). The oligodendrocytes produced upon TSA treatment were not only reduced in number but also demonstrated a less elaborate morphology, as has previously been shown upon *in vitro* differentiation of oligodendrocyte precursors [43].

**Figure 2:**
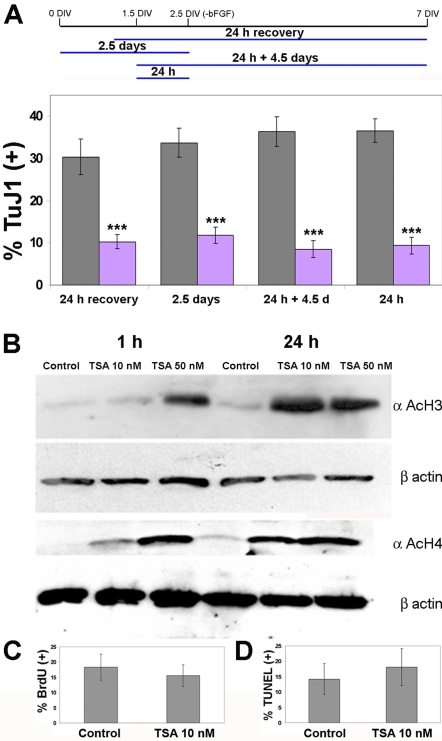
A 24-hour inhibition of HDACs before mitogen withdrawal is sufficient to suppress neurogenesis, independently of changes in proliferation and apoptosis, in differentiating neural progenitor cultures derived from embryonic GE. (A) Dissociated neurospheres were plated onto coverslips, and at 2.5 DIV bFGF was withdrawn (-bFGF), followed by 4.5 days of differentiation. Neurogenesis was evaluated at 7 DIV. Neurosphere cultures were treated for the indicated time periods (lines under the time line) with 10 nM TSA, then stained with the TuJ1 antibody to detect newborn neurons. DAPI staining of nuclei was used to count cells, and the percentage of neurons formed is shown. Mean values +/− SEM (n>3). *** = p<0.001, Mann-Whitney U test. (B) Western blot analysis of dissociated neurosphere cultures derived from embryonic GE treated with 10 nM TSA for 1 or 24 hours before bFGF withdrawal show a marked increase in acetylation levels of histone H3 and H4, using an antibody specifically recognizing acetylated lysine residues on the amino termini of histone H3 and H4. Loading levels were confirmed by reprobing each blot with an antibody recognizing β-actin (below each respective anti- acetylated histone panel). (C) Dissociated neurospheres were treated with BrdU (10 µM) alone or with 10 nM TSA and BrdU for 24 hours. Cells were then fixed and stained with an anti-BrdU antibody. DAPI staining was used to count cells, and the percentage of BrdU-positive cells is shown (n = 3). (D) After treatment of dissociated neurosphere cultures for 24 hours with 10 nM TSA, bFGF was withdrawn at 2.5 days *in vitro*, and apoptosis rates were measured one day later using the TUNEL assay. DAPI staining was used to count cells, and the percentage of TUNEL-positive cells is shown (n = 3).

Inhibition of HDACs for 7 days might lead to a large number of transcriptional changes in neural precursor cells. Since TSA can affect histone acetylation levels [57] and gene transcription [62] already several hours after application, we determined the critical time window for inhibition of HDAC function in order to inhibit neurogenesis in cultures derived from GE. We tested 4 different treatment periods (Fig. 2A): 1) 10 nM TSA applied first 24 hours after cells were plated out, to eliminate the possibility that TSA inhibits cells from adhering. 2) 2.5 days treatment of 10 nM TSA, from the time that cells are plated out until bFGF is withdrawn. 3) 10 nM TSA applied 24 hours before bFGF withdrawal and for a further 3.5 days until cells were fixed. 4) 10 nM TSA added only 24 hours before bFGF withdrawal. All 4 of these treatments resulted in a decrease of neurogenesis similar in magnitude to continuous treatment over 7 days. Importantly, only a 24-hour treatment of TSA before bFGF was withdrawn was necessary to reduce neurogenesis from 36% to 9%. This reduction in neurogenesis was accompanied with an increase of nestin (+) precursors (Control: 23.7±1.8%; 10 nM TSA : 41.2±13.5%) and GFAP (+) astrocytes (Control: 18.3±8.1%; 10 nM TSA: 32.0±2.0%, n = 2), as seen before (Fig. S3A, C and Fig. 1D, E, respectively). Interestingly, 10 nM TSA treatment after bFGF withdrawal did not significantly affect neurogenesis (data not shown).

To assess the acetylation levels of core histones in response to TSA, we employed antibodies specifically recognizing the acetylated forms of histone H3 and H4, using Western blots upon protein lysates. Application of 10 and 50 nM TSA to neurosphere-derived cultures one day before bFGF-withdrawal resulted in a detectable increase in acetylation levels of both histone H3 and H4 already within one hour, with an even higher increase after 24 hours (Fig. 2B).

TSA is known to inhibit cell cycle progression at G1 and G2 phases [63]. In order to assess the effects that TSA may have upon the cell cycle, we used BrdU labeling to assess the number of cells in the culture that had replicated their DNA. Dissociated neurospheres were treated with BrdU (10 µM) alone or with 10 nM TSA and BrdU for 24 hours. Cells were then fixed and stained with an anti-BrdU antibody. No significant differences were observed between control and TSA-treated cultures (Fig. 2C).

TSA can induce apoptosis in cancer cell lines and tumors, and synthetic analogues of TSA are employed in cancer therapy [64]. We addressed the possibility that neurons were being produced upon TSA treatment but then dying. Visual inspection of 10 nM TSA-treated cultures did not indicate an increase in cell death, and a DAPI staining of the nuclei of TSA-treated cells did not reveal an increase in pyknotic nuclei (Control: 6.9±1.4%, TSA: 5.9±0.8%, n = 4). We next examined apoptosis levels with a TUNEL assay. After treatment of cultures for 24 hours with 10 nM TSA, bFGF was withdrawn at 2.5 days *in vitro*, and apoptosis rates were measured one day later using the TUNEL assay. The percent of TUNEL (+) apoptotic cells was similar in control condition and upon TSA treatment (Fig. 2D).

### HDACs downregulate BMP2/4 signaling to promote GE neurogenesis *in vitro*

**Figure 3:**
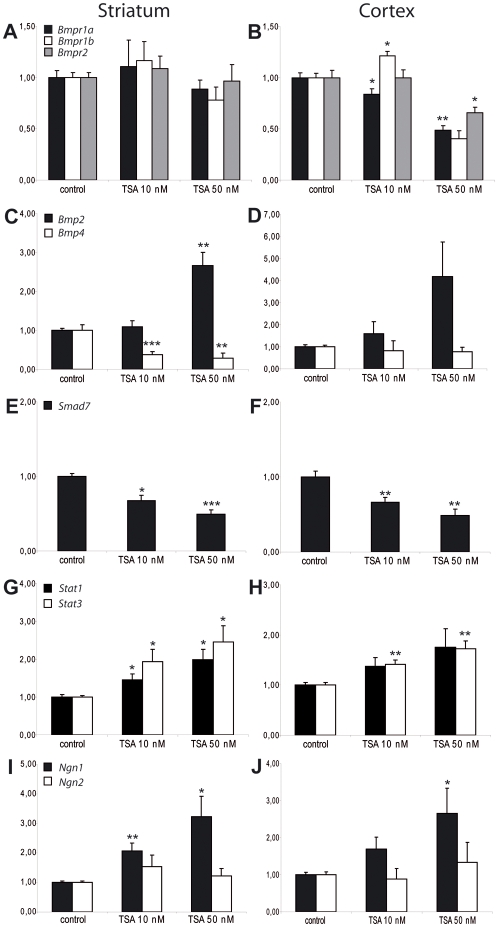
HDACs control expression of genes in the BMP2/4 signaling pathway and regulatory genes involved in neurogenesis and astrogliogenesis. (A–J) Quantitative real time RT-PCR was performed upon mRNA extracted from TSA-treated neurosphere cultures, comparing gene expression levels to untreated cultures. Dissociated neurosphere cultures derived from embryonic GE (A, C, E, G, I) or cortex (B, D, F, H, J) were plated out onto 10-cm dishes and treated 1.5 days later with vehicle (0.008% ethanol) or TSA (10 or 50 nM). RNA was extracted 12 hours later, and reverse-transcribed cDNA was analyzed using TaqMan probes recognizing the following genes: (A, B) *Bmpr1a*, *Bmpr1b*, *Bmpr2*; (C, D) *Bmp2*, *Bmp4*; (E, F) *Smad7*; (G, H) *Stat1*, *Stat3*; (I, J) *Ngn1*, *Ngn2*. cDNA was normalized using probes for GAPDH and HPRT. Mean values +/− SEM (n = 4–8). * = p<0.05, ** = p<0.01, *** = p<0.001, Student's T-test. (D) p = 0.06 for *Bmp2* expression increase in cortex treated with 50 nM TSA.

**Figure 4:**
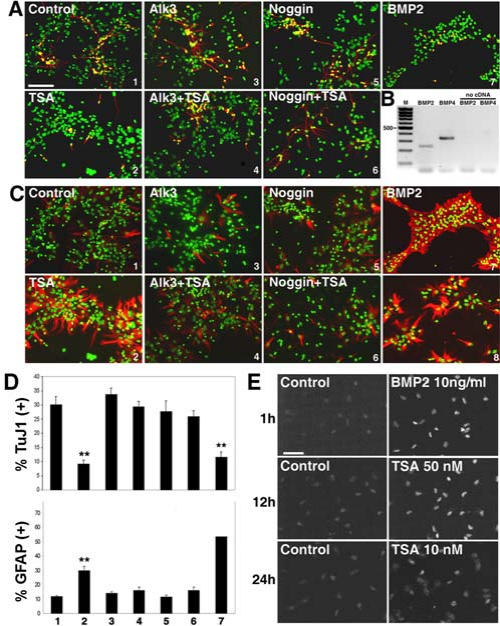
TSA-induced inhibition of neurogenesis from GE-derived precursors is restored by inhibition of the BMP2/4 signaling pathway. (A) Dissociated neurosphere cultures were plated out onto coverslips and treated 1.5 days later with either BMP2 (10 ng/ml), the BMP2/4 inhibitors Alk3-ECD (250 ng/ml) or Noggin (250 ng/ml), alone or in combination with TSA (10 nM). All growth factors, inhibitors, and bFGF were withdrawn 24 hours later. Cells were then cultured for an additional 4.5 days and analyzed by immunofluorescence, staining with the TuJ1 antibody (red) to detect neurons, and DAPI (green) to stain cell nuclei. Scale bar = 100 µm. (B) RT-PCR analysis of *Bmp2* and *Bmp4* expression in dissociated neurosphere cultures. Control PCR reactions lacking template cDNA are to the right. A 100-bp marker (M) with an indicated 500-bp band is to the left. (C) The identical cultures shown in (A) were stained with an anti-GFAP antibody (red) to detect astrocytes, and DAPI (green) to stain cell nuclei. Panel number 8 shows a culture from a separate experiment in which TSA was withdrawn at 7 DIV after 5 days of treatment and then cultured for an additional 4 days. (A, C) The numbers in the lower right corners of the figures correspond to the numbers beneath the graphs in (D). (D) Mean values +/− SEM (n>5). ** = p<0.01, Mann-Whitney U test. (E) Dissociated neurospheres were plated onto coverslips and were treated with BMP2 (10 ng/ml), or TSA (10 nM or 50 nM) after 1.5 days of *in-vitro* differentiation, and fixed after 1, 12, and 24 hours. Cells were stained with an antibody recognizing phosphorylated-Smad1/5/8. Displayed here are control conditions (left panels) and treatments (right panels, with indicated compound and concentrations). Scale bar = 50 µm.

To elucidate the molecular mechanism by which HDACs regulate neurogenesis, we investigated various developmental pathways known to control neurogenesis *in vivo*. We first examined the BMP2/4 signal transduction pathway. BMP2, BMP4, and their receptors are expressed in neurosphere cultures [14] and have been shown to promote astrocytic differentiation *in vitro* [14] and *in vivo* [15]. BMP2 is highly similar to BMP4 (93% amino acid identity), activates the same signaling pathway as BMP4, is also expressed in the subventricular zone of embryonic striatum [14], and like BMP4 was found to be expressed in differentiating neural stem cell cultures (Fig. 3C, D, 4B).

As we expected HDACs to act upon a transcriptional level to affect neurogenesis and astrogliogenesis in GE, we performed quantitative, real-time RT-PCR upon mRNA extracted from TSA-treated neurosphere cultures, comparing gene expression levels to untreated cultures. 12 hours after treatment of 1.5 DIV cultures with TSA, the expression of the BMP2/4-specific receptors *Bmpr1a*, *Bmpr 1b*, and *Bmpr2* were unchanged in GE-derived cultures (Fig. 3A). In contrast, the expression of *Bmp2* was upregulated dramatically in GE-derived cultures (Fig. 3C) after 12 hours of TSA treatment, whereas *Bmp4* was downregulated (Fig. 3C). We next examined the expression of the inhibitory factor *Smad7* [55] in our cultures. *Smad7* expression was downregulated by TSA application at both concentrations (Fig. 3E). In addition, the expression of the astrogliogenesis-promoting transcription factors *Stat1* and *Stat3* was upregulated by TSA-treatment (Fig. 3G). Curiously, the expression of the neurogenesis-promoting transcription factor *Ngn1* was also upregulated by TSA-treatment, while that of *Ngn2* remained unaffected (Fig. 3I). All of these effects showed a TSA dose-dependence (Fig. 3C, E, G, I).

In order to test the relevance of the transcriptional control of *Bmp2* and *Smad7* gene expression by HDACs, we decided to influence the BMP2/4 signaling pathway by boosting or inhibiting the extracellular signal. Recombinant BMP2 was applied to differentiating neurosphere-derived cultures one day before the withdrawal of bFGF at concentrations ranging from 10–100 ng/ml. This resulted in a dramatic effect, in that over 50% of the cells differentiated into astrocytes, as previously reported (Fig. 4C, D; [14]). The GFAP+ cells were clustered together in what resembled astrocytic “islands” (Fig. 4C, panel 7). We also observed similar clusters of GFAP+ cells in cultures in which 10 nM TSA was withdrawn after 5 days of treatment and then cultured for an additional 3 to 4 days, for a total of 10–11 days (Fig. 4C, panel 8). In order to test the involvement of endogenous BMP signaling in normal and TSA-treated cultures, cells were treated with two different BMP2/4 inhibitors. Alk3-ECD is a recombinant protein consisting of the extracellular domain of the BMPR1A receptor that can bind to BMP2 and -4 with high affinity, preventing them from binding to the endogenous BMPR1A receptor [65]. Noggin is a secreted polypeptide that binds to BMPs and prevent their binding to and activation of their receptors [66]. Each inhibitor was added for 24 hours before bFGF withdrawal, with or without 10 nM TSA, and then removed when bFGF was withdrawn. The cultures were analyzed 4.5 days later, at 7 DIV. The use of either Alk3-ECD or noggin (each at a concentration of 250 ng/ml) alone did not affect neurogenesis (Fig. 4A, D) or astrocyte production (Fig. 4C, D). However, addition of either BMP2/4 inhibitor in combination with 10 nM TSA completely restored normal levels of neurogenesis (Fig. 4A, D). In addition, the production of astrocytes was reduced to levels seen in untreated cells (Fig. 4C, D). Longer treatment of cultures with these reagents, starting 24 hours before bFGF withdrawal but continuing for an additional 4.5 days, produced similar results (Neurogenesis: Control: 31.1±0.7%; TSA: 8.2±1.1%; Alk3-ECD: 30.4±1.1%; Alk-3 & TSA: 27.9±1.4%; Noggin: 30.0±3.9%; Noggin & TSA: 24.8±2.3%, n = 2, reagent concentrations as above).

In order to investigate the effect of TSA upon neural stem cell cultures, we next examined the activation of the BMP2/4 signaling pathway. Binding of BMP2/4 to their cognate receptors results in the phosphorylation of two adjacent serine residues in the Smad1/5/8 proteins, followed by their translocation to the nucleus where they act as transcriptional regulators of BMP2/4-responsive genes [67]. We examined the cultures with an antibody that specifically recognizes Smad1/5/8 proteins phosphorylated at these serine residues, staining cultures 1, 4, 12, and 24 hours after reagents were added (Fig. 4E). Addition of 10 ng/ml BMP2 to cultures 24 hours before bFGF withdrawal caused an accumulation of phosphorylated Smad1/5/8 in the nucleus after just 1 hour of treatment (Fig. 4E). This localization was sustained at 4 hours, whereas by 12 and 24 hours it was no longer detectable. A nuclear localization of phosphorylated Smad1/5/8 became visible after 12 hours of treatment with 50 nM TSA and after 24 hours of treatment with 10 nM TSA (Fig. 4E). This delay in Smad1/5/8 nuclear localization compared to BMP2 application may reflect the need to induce a change in transcription patterns of genes involved in BMP signaling, such as *Bmp2* (Fig. 3C) itself or *Smad7* (Fig. 3E).

Additionally, we investigated two other pathways that are known to regulate neurogenesis in mammals and that had been implicated in the inhibition of retinal neurogenesis in a mutation in the zebrafish *hdac1* gene [46]. Examination by Western blot of the protein levels of the intracellular domain of Notch1, representing the cleaved, activated domain [68], revealed no change in its expression upon a 7-day treatment with 10 nM TSA, comparing cultures at 1, 2.5, 3.5, and 7 DIV (Fig. S4A). To examine a possible involvement of the Wnt signaling pathway, we treated neurosphere-derived cultures with inhibitors of Wnt signaling, Dkk1 (0.5 µg/ml) and sFRP2 (0.2 µg/ml). These inhibitors were added 24 hours before bFGF withdrawal, and neurogenesis examined after a further 4.5 days of culture. In the presence of the two inhibitors, no changes were seen in neurogenesis, in comparison with no TSA or with 10 nM TSA treatment, respectively (Fig. S4B). Although neither of these experiments definitively rule out the involvement of Notch or Wnt signaling in HDAC-mediated neurogenesis, the full complementation of neurogenesis seen with BMP2/4 inhibitors in combination with TSA treatment suggests that this is the most important neurogenic pathway controlled by HDACs.

### HDACs inhibit cortical neurogenesis *in vitro* through a BMP2/4-dependent pathway

**Figure 5:**
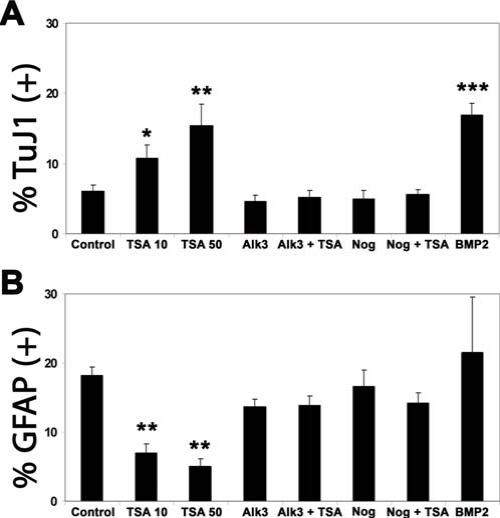
HDAC inhibition by TSA treatment promotes neurogenesis and inhibits astrogliogenesis in a BMP2/4-dependent manner in differentiating neural progenitor cultures derived from embryonic cortex. Dissociated neurosphere cultures derived from 15.5 d.p.c. cortex were plated onto coverslips and treated 1.5 days later with either BMP2 (10 ng/ml), the BMP2/4 inhibitors Alk3-ECD (250 ng/ml) or Noggin (250 ng/ml), alone or in combination with TSA (10 nM). All inhibitors and bFGF were withdrawn 24 hours later. Cells were then cultured for an additional 4.5 days and analyzed by immunofluorescence, using either the TuJ1 antibody to detect neurons (A) or an anti-GFAP antibody to detect mature astrocytes (B). DAPI staining of nuclei was used to count cells, and the percentage of neurons (A) and astrocytes (B) formed is shown. Mean values +/− SEM (n = 3). * = p<0.05, ** = p<0.01, *** = p<0.001, Mann-Whitney U test.

Previous reports have described differing effects of BMP2/4 signaling upon neurogenesis in different parts of the developing brain, in that these growth factors seem to promote neurogenesis in embryonic cortex [20], [21] but inhibit neurogenesis in GE [14]. In order to compare the effect of HDAC inhibition upon cultures prepared from GE, we performed *in vitro* differentiation upon precursor cells derived from embryonic cortex. Neurosphere cultures were prepared from C57BL/6J embryonic cortex at 15.5 d.p.c., cultured for seven days as floating cell aggregates, dissociated mechanically, and then differentiated upon coverslips using conditions identical to those used for GE-derived cultures. 10 nM TSA was added to the medium upon plating out of dissociated neurospheres and again after 2.5 days when basic FGF was withdrawn (Fig. 1A). After an additional 4.5 days cells were fixed and stained with the TuJ1 antibody to detect β-tubulin III expression. In contrast to GE-derived cultures, the percentage of neurons increased from 6.0±0.9% in untreated cultures to 10.8±1.9% upon TSA treatment (Fig. 5A), evaluating cell number by counting DAPI-stained nuclei. In contrast, astrocytic differentiation was reduced in TSA-treated cells, changing from 18.0±1.3% in untreated cells to 6.9±1.4% in TSA-treated cells (Fig. 5B).

In cortical cultures treated with TSA, similar transcriptional effects upon members of the BMP2/4 signaling pathway were observed as in cultures derived from GE. In both culture types, 10 and 50 nM TSA treatment resulted in an increase in *Bmp2* (Fig. 3C, D), *Stat1*, *Stat3* (Fig. 3G, H), and *Ngn1* (Fig. 3I, J), and a decrease in *Smad7* mRNA levels (Fig. 3E, F). In contrast to GE-derived cultures, all three BMP2/4 receptors showed a reduction in expression in cortical cultures at 50 nM TSA (Fig. 3B). In order to determine if BMP2/4 signaling was also involved in the TSA-mediated changes in neurogenesis and astrogliogenesis in the cortex, recombinant BMP2 (10 ng/ml) was applied to differentiating cortex-derived cultures one day before the withdrawal of bFGF. In contrast to GE-derived cultures, BMP2 was able to promote neurogenesis to the same extent that 50 nM TSA application had (Fig. 5A). Interestingly, BMP2 treatment did not lead to a statistically significant increase in astrogliogenesis, in contrast to the GE-derived cultures (Fig. 5B). In order to test the involvement of endogenous BMP2/4 signaling, cells were treated with the extracellular BMP2/4 inhibitors Alk3-ECD and noggin. Each inhibitor was added for 24 hours before bFGF withdrawal, with or without 10 nM TSA application, and was removed when bFGF was withdrawn. The cultures were analyzed 4.5 days later, at 7 DIV. As was seen in GE-derived cultures, neither Alk3-ECD nor noggin alone influenced neurogenesis (Fig. 5A) or astrogliogenesis (Fig. 5B). However, addition of either of these BMP inhibitors in combination with TSA restored neurogenesis to levels seen in the control samples (Fig. 5A). Again, as seen in GE, astrogliogenesis was also returned to control levels upon treatment with both TSA and either inhibitor (Fig. 5B).

### Bidirectional control of neurogenesis by HDACs *in vivo*

**Figure 6:**
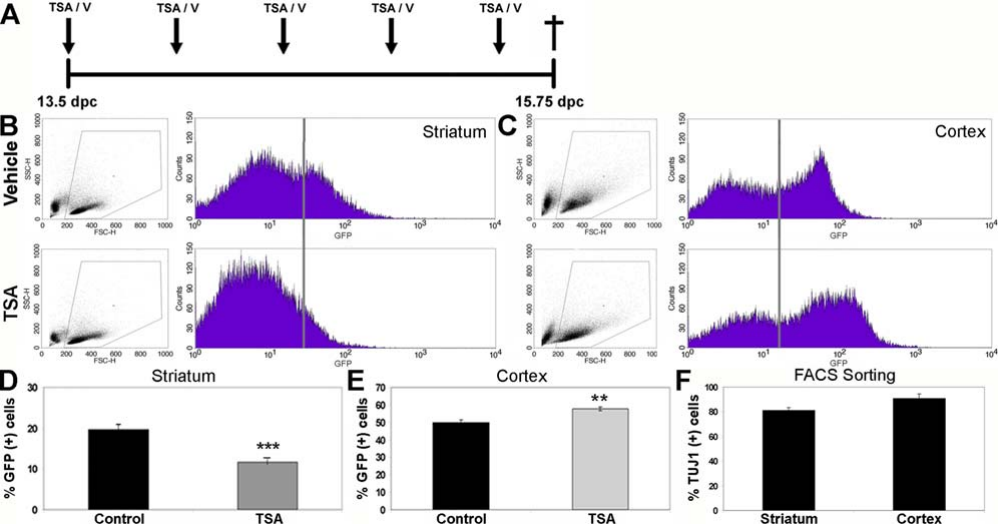
HDAC inhibition by TSA *in utero* restricts GE-derived neurogenesis and promotes cortical neurogenesis. (A) Timed-pregnant female *tauGFP* mice were injected every 12 hours with vehicle (B, C, upper diagrams) or with 12.5 µg TSA (B, C, lower diagrams), starting from 13.5 d.p.c., and embryos were examined 54 hours later at 15.75 d.p.c. FACS analysis was performed upon dissociated GE-derived (B) and cortical (C) cells, using GFP as a marker for newborn neurons. For each treatment and tissue (B, C), a scatter plot showing forward (FSC-H) versus side scatter (SSC-H) is shown on the left, and a histogram displaying GFP intensity versus the number of events is shown on the right. The population of cells analysed for green fluorescence is indicated by the gate in the scatter plots (left panel). All cells to the right of the vertical line in the histogram (right panel) were judged to be GFP-positive and therefore neurons. (D, E) Quantitation of the GFP-positive neuronal population in the developing GE (D) and cortex (E), treated with vehicle (Control) or with TSA (TSA). Mean values +/− SEM (n>6). ** = p<0.01, *** = p<0.001, Mann-Whitney U test. (F) GFP-positive cells were collected from embryonic cortex and GE by FACS sorting, plated out, and cultured for 3 hours, and then stained for expression of β-tubulin III to verify their neuronal identity (n = 2).

In order to determine whether the HDAC-mediated modulation of neurogenesis and astrogliogenesis observed *in vitro* is of physiological relevance, TSA was employed to inhibit HDACs during embryogenesis *in vivo*. Timed-pregnant C57BL/6J female mice were injected every 12 hours with vehicle (8% ethanol in 1× PBS) or with 12.5 µg TSA in vehicle, an amount of TSA that had been previously shown to be nontoxic to murine embryos [69]. Injections began at 13.5 days *post coitum* (d.p.c.), a time point when neurogenesis has already begun in GE and cortex, and embryos were examined two days later (Fig. 6A). In order to quantify neurogenesis, we performed FACS analysis upon dissociated cortical and GE-derived cells from TSA-injected mice of the *tauGFP* line, in which the cDNA encoding EGFP has been inserted at the tau locus [60]. In this line, EGFP is expressed at high levels in all neurons shortly after their birth in a time frame similar to induction of β-tubulin III, and both markers can therefore be used for the quantification of neurogenesis [70]. As judged by the fraction of cells that were GFP-positive, neurogenesis in GE was reduced by 45% (Fig. 6B, D), whereas neurogenesis increased in the cortex modestly by 10% (Fig. 6C, E). The validity of FACS-sorting of the cortical and GE-derived cells was subsequently confirmed. Cells were collected with the same gating conditions that were used for the determination of the neuronal fraction of the populations, and the GFP-positive populations were examined by staining for TuJ1, a standard marker for newborn neurons that recognizes β tubulin III [71]. In the cortex and GE, over 90% and 80%, respectively, of the cells judged by FACS to be GFP-positive were found to be TuJ1 positive (Fig. 6F). TuJ1 staining of acutely-dissociated cortex and GE from embryos exposed to TSA confirmed the FACS results (data not shown).

**Figure 7:**
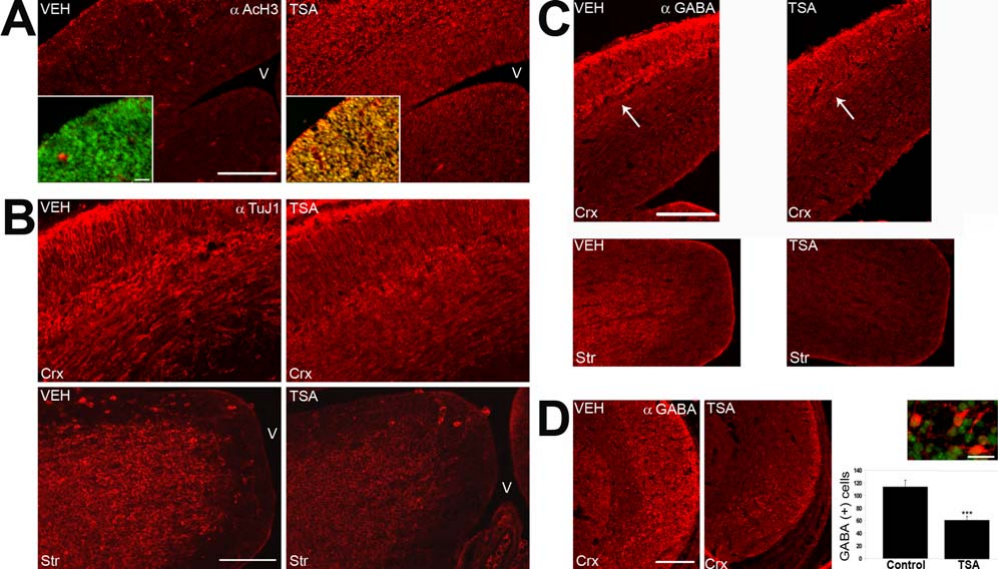
HDAC inhibition by TSA *in utero* results in a decrease of neurogenesis in the GE and a corresponding reduction in GE-derived GABA-positive cells undergoing tangential migration in the cortex. Timed-pregnant *tauGFP* mice were injected every 12 hours with vehicle (VEH) or with TSA (TSA) starting from 13.5 d.p.c., and embryos were examined at 15.75 d.p.c., using immunohistofluorescence with the indicated antibodies (upper right). In all panels, dorsal is at the top. Lateral is to the left (A, B, C) or the right (D) of each panel, respectively, and “V” indicates the ventricle. (A) Both cortex (upper portion of figure and inset) and GE (lower portion of figure to the right of the inset) show an increase in the acetylation of histone H3 upon TSA treatment, as revealed with an anti-acetylated-histone H3 antibody. Inset: red = anti-acetylated-histone H3 antibody, green = DAPI. (B) Using the TuJ1 antibody to detect newborn neurons, the GE (Str) shows a decrease in signal in TSA-treated embryos, reflecting a decrease in neurogenesis, while the cortex (Crx) looks essentially the same upon TSA treatment. (C) An antibody recognizing GABA labels GE-derived interneurons migrating tangentially in the intermediate zone of the cortex (Crx, arrow). This migrating population is substantially decreased in TSA-treated embryos. GABA-positive cells are also substantially decreased in GE (Str). (D) The number of GABA-positive cells (photos left, inset: red = anti-GABA, green = DAPI) were quantified in the ventrolateral cortex (Crx), reflecting the number of cells per slide in one hemisphere (graph: Mean values +/− SEM (n = 3). *** = p<0.001, Student's T-test. Scale bars: (A, C, D) 200 µm, (B) 100 µm, (A: inset) 25 µm, (D: inset) 20 µm.

**Figure 8:**
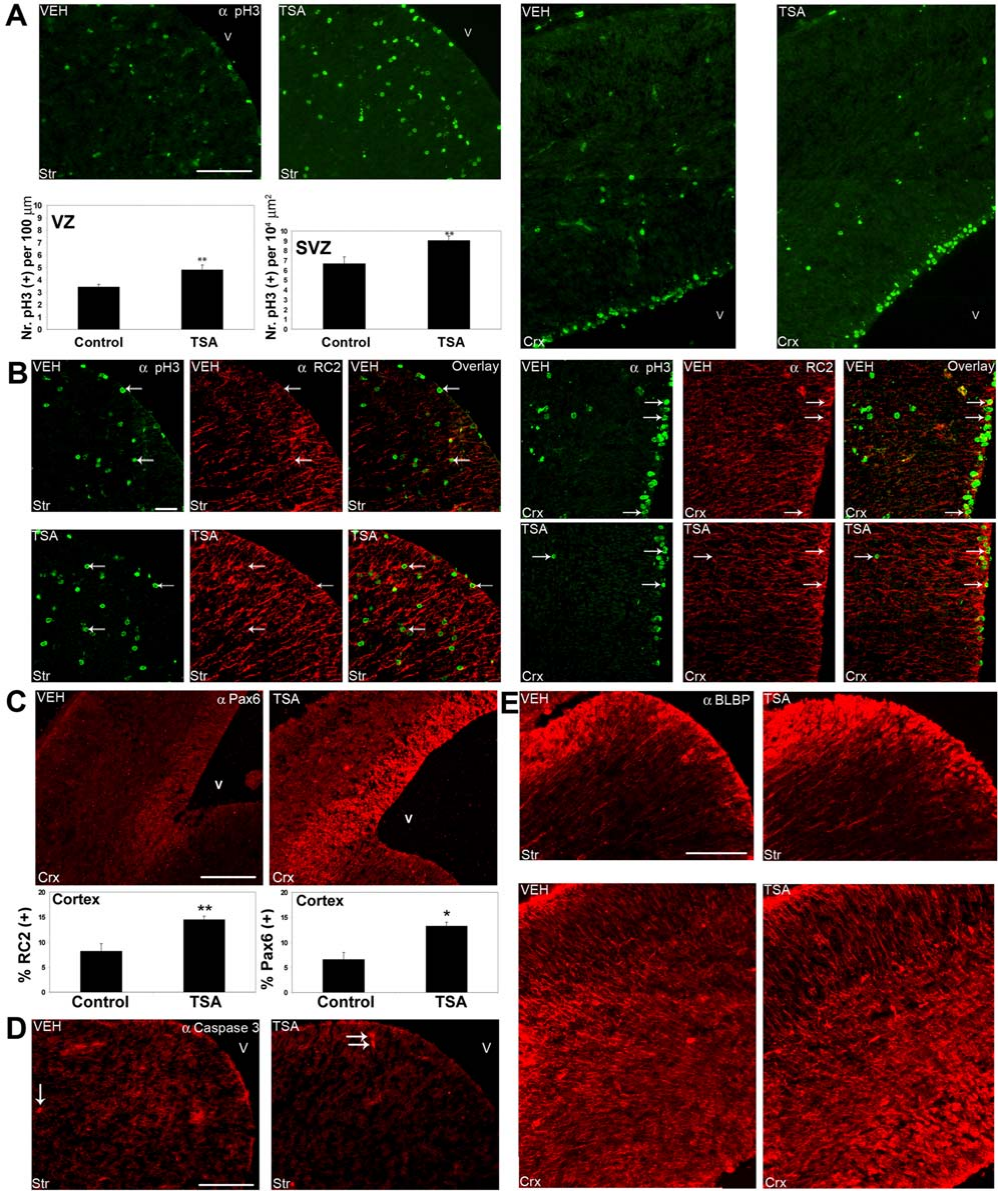
HDAC inhibition by TSA *in utero* results in an increase in proliferation in the ventricular (VZ) and subventricular zones (SVZ) in embryonic GE, and an increase in the number of radial glia in the cortex. Timed-pregnant *tauGFP* mice were injected every 12 hours with vehicle (VEH) or with TSA (TSA) starting from 13.5 d.p.c., and embryos were examined at 15.75 d.p.c., using immunohistofluorescence with the indicated antibodies (upper right). (A) An antibody detecting phosphorylated-histone H3 (green) was used to detect mitotic cells, and TSA can be seen to increase mitotic figures in the ventricular and subventricular zones of GE (Str, left) but not in the cortex (Crx, right). In order to quantify phosphorylated-histone H3-positive cells, the VZ was delimited with a 30 µm line normal to the ventricle, and all positive cells within this area were counted and displayed as the number of cells within this area along the length of the ventricular surface (per 100 µm). SVZ cells were counted as those cells lying between 30 and 200 µm basal to the ventricular surface, and displayed as the number of cells within this area (per 10^4^ µm^2^). This quantitation is shown (below photos). (B) The RC2 antibody was used to label neural precursors / radial glia, and an increase in signal can be seen in TSA-treated embryos in both the cortex (Crx) and GE (Str) (middle panels, red). Many of the RC2-positive cells were identified as mitotic by phospho-histone H3 staining (left panels, green, arrows), as indicated by an overlap of the two images (right panels, arrows) (C, E) A similar increase was seen using an antibody recognizing the radial glial markers Pax6 (C, red) or BLBP (E, red). (C, graphs) The percentage of cells expressing RC2 (left) or Pax6 (right) in the cortex was quantitated by staining acutely dissociated cultures with the respective antibodies, using a nuclear DAPI stain to evaluate total cell number. (D) An antibody detecting cleaved caspase 3 recognizes very few positive nuclei (red, arrows) in GE of either vehicle- or TSA-treated embryos. In all panels, dorsal is at the top, lateral is to the left, and “V” marks the ventricle. Scale bars: (A, D, E) 100 µm, (B) 50 µm, (C) 200 µm. (C) Mean values +/− SEM (n>3). * = p<0.05, ** = p<0.01, Mann-Whitney U test.

We next examined the influence upon telencephalic development of HDAC inhibition, using immunohistofluorescence upon embryos after one or two days of injections starting at 13.5 d.p.c. Inspection of the entire brain revealed no apparent anatomical abnormalities. As expected, acetylation of histone H3 increased strongly in cells throughout the cortex and GE upon TSA treatment (Fig. 7A), using an antibody that specifically recognizes acetylated-histone H3. Using TuJ1 as a marker for newborn neurons, we could clearly observe a decrease in signal in GE, reflecting the decrease in neurogenesis seen with FACS analysis (Fig. 7B). Many of the newborn neurons in the medial GE start migrating tangentially into the cortex, using a route through the cortical intermediate zone [8]. Using an antibody against GABA to label these migrating inhibitory interneurons, we were clearly able to see a reduction in staining of both the GE and also the cortical intermediate and marginal zones in TSA-treated embryos, reflective of a decrease in neurogenesis in the GE (Fig. 7C). To quantitate this decrease, we counted the number of GABA-positive neurons in the ventrolateral cortex (at the boundary with the mantle zone of the lateral GE), where individual GABA-positive neurons are easy to identify. We observed a 50% reduction in the numbers of GABA-positive neurons in TSA-treated cultures, corresponding to the overall decrease in neurogenesis seen with FACS analysis and TuJ1 staining (Fig. 7D). This decrease in GE-derived neurons was not caused by an increase in apoptosis of precursors or newborn neurons, as we observed very few apoptotic figures in either vehicle- or TSA-injected GE or the cortex at 15.75 d.p.c, as judged by immunolabeling of activated caspase 3 (Fig. 8D). As would be expected for the modest increase in cortical neurogenesis revealed by FACS analysis (Fig. 6C, E), no obvious change in TuJ1 staining could be observed in the cortex (Fig. 7B).

Interestingly, the number of mitotic cells increased both in the ventricular (VZ) and subventricular zones (SVZ) of GE, but not in the VZ / SVZ of the cortex (Fig. 8A), as judged by the use of an antibody recognizing phosphorylated-histone H3, which becomes phosphorylated specifically during mitosis [72]. In order to quantify phosphorylated-histone H3-positive cells, the VZ was delimited with a 30 µm line normal to the ventricle, and all positive cells within this area were counted and displayed as the number of cells within this area along the length of the ventricular surface (per 100 µm, Fig. 8A). SVZ cells were counted as those cells lying between 30 and 200 µm basal to the ventricular surface, and displayed as the number of cells within this area (per 10^4^ µm^2^, Fig. 8A).To identify the cells whose proliferation was increasing as a result of TSA treatment, markers for radial glial cells were examined, as radial glia are known to be precursors for both neurons as well as astrocytes in the developing cortex and GE [2], [3]. An RC2 antibody, which detects neural precursors and radial glia during embryogenesis [73], revealed a distinct upregulation upon TSA treatment in both GE and the cortex (Fig. 8B). Similarly, we observed an increase in the staining intensity of the transcription factor Pax6 in the cortex of TSA-treated animals, most prominently in the ventromedial cortex (Fig. 8C), and an increase in the staining intensity of BLBP, another marker for radial glia, in both GE and cortex after TSA treatment (Fig. 8E). In order to test if these changes reflected an increase in the numbers of radial glia in the TSA-treated embryos, or just an increase in the expression level in cells already expressing these markers, we plated out acutely-dissociated cells from control and TSA-treated embryos and fixed them after 2 hours, followed by a subsequent staining with the corresponding markers. Here, we clearly saw an increase in the number of cells staining positive for both RC2 and Pax6 in the cortex (Fig. 8C), with no change in GE (data not shown).

**Figure 9:**
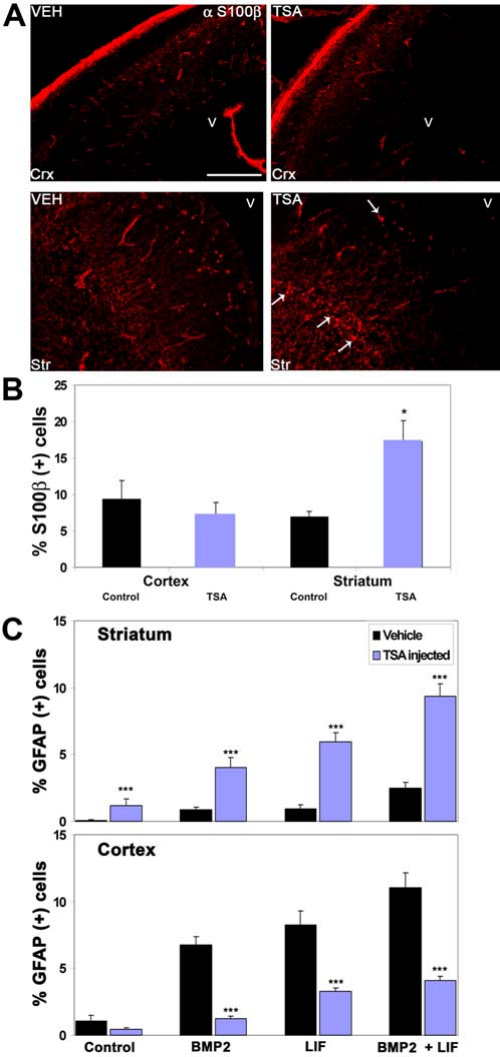
HDAC inhibition by TSA *in utero* potentiates premature astrogliogenesis in embryonic GE. Timed-pregnant *tauGFP* mice were injected every 12 hours with vehicle (VEH) or with TSA (TSA) starting from 13.5 d.p.c., and embryos were examined at 15.75 d.p.c. (A) An anti S100-β antibody (red) demonstrates a precocious production of immature astrocytes in GE (Str, arrows) but not the cortex (Crx) of TSA-treated embryos. In all panels, dorsal is at the top, lateral is to the left, and “V” marks the ventricle. Scale bar: 200 µm. (B) Quantification performed upon stainings of acutely-dissociated cultures prepared from GE and cortex of 15.75 d.p.c. embryos indicated an increase in the number of S100-β-positive cells from GE but not the cortex. (C) Potentiation of astrogliogenesis in GE was revealed by the culture of acutely dissociated cells from GE and cortex, respectively, of 15.75 d.p.c. vehicle- and TSA-treated embryos. Dissociated cultures were treated for 2 days with the astrocyte-promoting factors BMP2 (10 ng/ml) and / or LIF (25 ng/ml). Cultures were fixed after an additional 5 days of culture and stained for astrogliogenesis with an antibody recognizing GFAP, using a DAPI stain to evaluate total cell number. (B, C) Mean values +/− SEM (n = 4). * = p<0.05, *** = p<0.01, Mann-Whitney U test, p values were all determined by comparison of control and TSA-treated for any given condition.

As had been seen *in vitro*, a reduction in neurogenesis in GE may be explained by a precocious astrogliogenesis, which had been previously seen in a genetic ablation of DNA methyltransferase 1 (*Dnmt1*) [74]. To examine this, an antibody recognizing S100β, a marker for newborn astrocytes, was used to examine TSA-treated embryos. An increase in staining could be observed specifically in GE (Fig. 9A), which was accompanied by an increase in the number of S100β-positive cells detected in acutely dissociated cultures (Fig. 9B). The high background in S100β (7–9%) staining came from endothelial cells of blood vessels, seen as tubular structures in Fig. 9A, whereas the increase in staining in GE of TSA-treated embryos is derived from small punctuate cells with one or two processes (Fig. 9A, arrows). Whether this change reflects the birth of newborn astrocytes was difficult to ascertain, as we could not confirm their identity *in vivo* with a stain for GFAP, a marker for more mature astrocytes (data not shown). In order to see if the *in situ* environment is prohibitive for the production of astrocytes at this time point, acutely-dissociated cultures were made from both GE and cortex of vehicle- and TSA-injected embryos. The freshly dissociated cultures were treated for 2 days with either BMP2 (10 ng/ml), LIF (25 ng/ml), or both factors, both of which have been previously shown to promote astrogliogenesis *in vitro* from embryonic brain precursors [12], [14]. The cultures were then examined five days later at 7 DIV, using an anti-GFAP antibody to detect newborn astrocytes. In both acutely-dissociated GE-derived and cortical cells, both BMP2 and LIF could promote the production of GFAP-positive astrocytes, and the combination of the two factors showed an even stronger effect, as reported previously [12] (Fig. 9C). However, in cultures derived from GE of TSA-treated embryos, astrogliogenesis was substantially promoted not only in the presence of LIF and BMP2 but also in their absence (Fig. 9C). In distinct contrast, BMP2 and LIF-promoted astrogliogenesis was significantly inhibited in cortical cultures derived from TSA-treated embryos (Fig. 9C). Therefore, HDAC inhibition showed the same effect upon astrogliogenesis from acute cultures derived from TSA-injected embryos as it did upon *in vitro*-differentiated neurosphere cultures prepared from these two brain regions.

**Figure 10:**
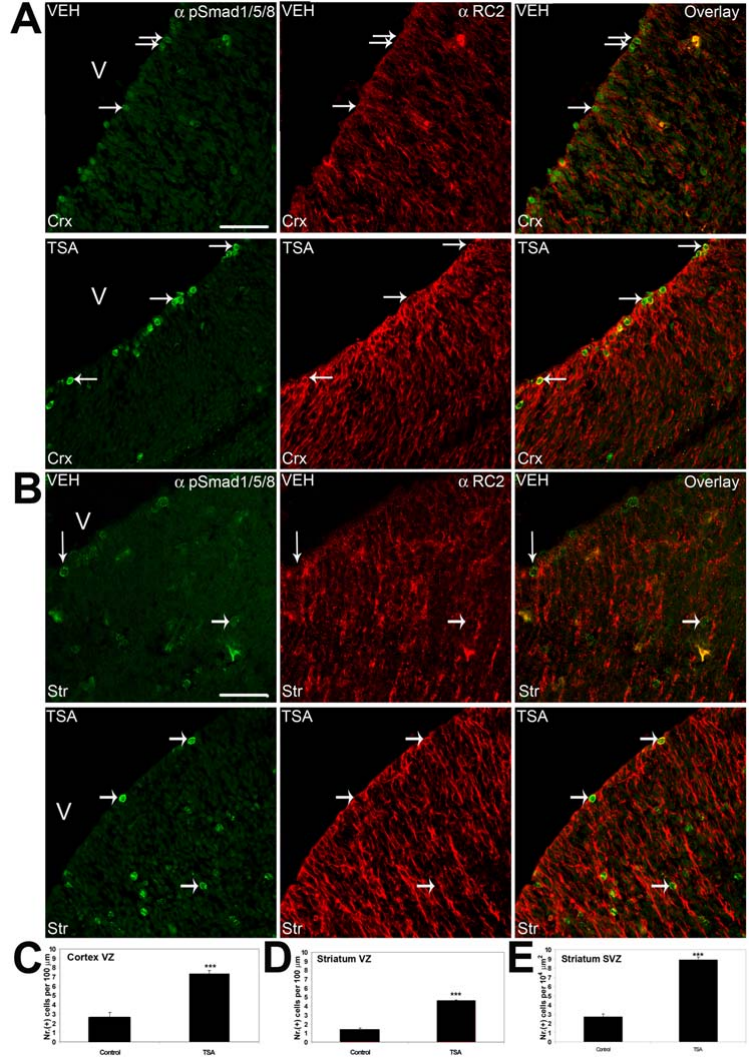
HDAC inhibition by TSA *in utero* promotes translocation of phosphorylated Smad1/5/8 to the nucleus of neural precursor / radial glial cells. Timed-pregnant *tauGFP* mice were injected every 12 hours with vehicle (VEH) or with TSA (TSA) starting from 13.5 d.p.c., and embryos were examined at 15.75 d.p.c., using immunohistofluorescence with the indicated antibodies (upper right). (A, B) An antibody recognizing phosphorylated-Smad1/5/8 (green, left panels) shows an increase in the nuclear localization of these transcription factors in the ventricular zone of the cortex (A) and the ventricular and subventricular zone of GE (B) upon treatment with TSA. Amost all of the cells showing nuclear phosphorylated-Smad1/5/8 were identified as radial glia by positive staining using the neural precursor / radial glial marker RC2 (red, middle panels, arrows), as indicated by an overlap of the two images (right panels, arrows). In all panels, dorsal is at the top, lateral is to the right, and “V” marks the ventricle. Scale bar: 50 µm. (C, D, E) Quantitation of cells positive for nuclear phosphorylated-Smad1/5/8 in the ventricular zone of the cortex (C), and the ventricular (D) and subventricular zone (E) of GE, after 2 days of TSA treatment, using a DAPI stain to quantitate cell number. For the purpose of counting phosphorylated-Smad1/5/8-positive cells in GE and cortex, the VZ and SVZ of the respective regions were defined as above for the phosphorylated-histone H3 stains (Figure 8A). Mean values +/− SEM (n = 5). *** = p<0.001, Mann-Whitney U test.

In order to see if the effects of HDAC inhibition upon neurogenesis and astrogliogenesis were occurring through a BMP2/4-dependent pathway, as demonstrated *in vitro*, the nuclear localization of phosphorylated Smad1/5/8 was examined in vehicle- and TSA-treated embryos. In both the cortex (Fig. 10A, arrows) and GE (Fig. 10B, arrows) of vehicle-treated embryos, faint staining could be seen in cells of the ventricular zone of the cortex and GE and the subventricular zone of GE. In contrast, nuclear localization of phosphorylated Smad1/5/8, as revealed by DAPI colocalization (data not shown), was increased in these regions in TSA-treated embryos (Fig. 10A, B, left panels). Quantitation of the cells with nuclear localization of phosphorylated Smad1/5/8 revealed a large increase in these three regions (Fig. 10C, D, E), using the same counting method as for phosphorylated-histone H3 (Fig. 8A). Most of the cells responding to TSA-treatment were also seen to be co-labelled for the neural precursor marker RC2 (Fig. 10A, B, middle and right panels), indicating that neural precursors / radial glia are the cell population responding to HDAC inhibition by nuclear localization of the downstream effector of BMP2/4 signaling, phosphorylated Smad1/5/8.

**Figure 11:**
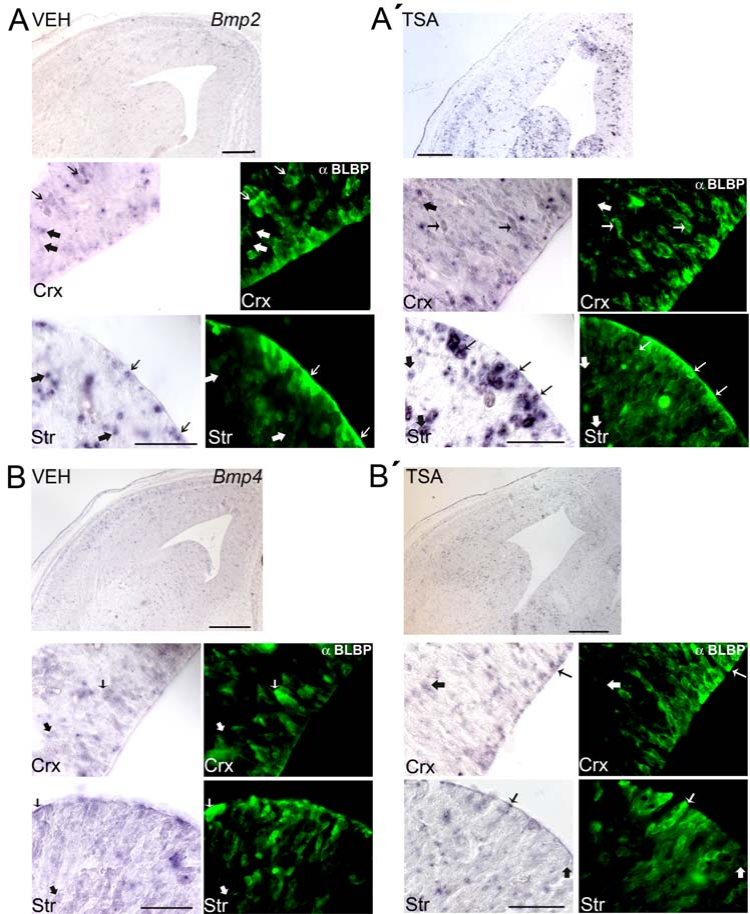
HDAC inhibition by TSA *in utero* promotes an upregulation in the expression of *Bmp2* but not *Bmp4* in both radial glial and non-radial glial cells of the embryonic cortex and GE. Timed-pregnant *tauGFP* mice were injected every 12 hours with vehicle (A, B: VEH) or with TSA (A′, B′: TSA) starting from 13.5 d.p.c., and embryos were examined at 14.5 d.p.c. using *in situ* hybridization for mRNA expression of *Bmp2* (A, A′: top and left panels) and *Bmp4* (B, B′: top and left panels), combined with immunohistofluorescence for the radial glial marker BLBP (A, A′, B, B′: green, right panels). The top photo in each panel (A, A′, B, B′) shows *Bmp2* or *Bmp4* expression in the cortex and GE, while the middle and bottom panels are magnifications of the cortex (Crx) and GE (Str), respectively. Each *in situ* hybridization (middle and bottom rows, left panel) is followed by a staining of this section for the radial glial marker BLBP (middle and bottom rows, right panel). Thin arrows indicate *Bmp2*- (A, A′) or *Bmp4*-expressing (B, B′) cells staining positive for BLBP, while thick arrows indicate *Bmp2*- (A, A′) or *Bmp4*-expressing (B, B′) cells negative for BLBP. In all panels, dorsal is at the top and lateral is to the left. Scale bars: (A, A′, B, B′: top row) 200 µm; (A, A′, B, B′: middle and bottom rows) 50 µm.

To see which cells were producing BMP2 and BMP4 in the brain, *in situ* hybridization was coupled with immunohistofluorescence for the radial glial marker BLBP (Fig. 11). As before, 13.5 d.p.c. timed-pregnant mice were injected every 12 hours with vehicle or with 12.5 µg TSA in vehicle, and embryos were examined 24 hours later. In vehicle-treated 14.5 d.p.c. embryos, low levels of *Bmp2* expression could be seen throughout the cortex and GE (Fig. 11A, top panel). At the ventricular zone of the cortex and the GE, some BLBP-positive radial glia were seen to express *Bmp2* (Fig. 11A, thin arrows, middle and bottom panels), but many *Bmp2*-expressing cells were not labeled with the BLBP-antibody, especially in the subventricular zones of GE and cortex (Fig. 11A, thick arrows, middle and bottom panels). Upon 24-hour treatment with TSA, a strong up-regulation of *Bmp2* could be observed in both GE and cortex (Fig. 11A′, top panel). Although some of the cells strongly-expressing *Bmp2* co-labelled for BLBP (Fig. 11A′, thin arrows, middle and bottom panels), most of them did not (Fig. 11A′, thick arrows, middle and bottom panels). In contrast, the expression pattern of *Bmp4* did not appear to change significantly upon TSA treatment (Cf. Fig. 11B with Fig. 11B′, labeled as above). Together, these data are in correlation with that seen in *in vitro* cultures, in which HDAC inhibition upregulated the mRNA expression of *Bmp2* but not of *Bmp4*. It also supports the idea that not only are radial glia expressing BMP2/4, but they are also responding to these factors, as seen with the nuclear localization of phosphorylated Smad1/5/8 after HDAC inhibition (Fig. 10).

## Discussion

We report here a requirement of HDAC activity for the generation of neurons from the GE of the embryonic mouse telencephalon and a modulatory role of HDACs in cortical neurogenesis. TSA, a potent and specific inhibitor of class I and II HDACs, was seen in the GE to greatly reduce neurogenesis *in vivo* and almost completely block neurogenesis *in vitro*. In contrast, in the cortex neurogenesis was actually enhanced upon HDAC inhibition. We have identified the BMP2/4 signaling pathway as the target of HDAC activity, both in the GE and the cortex. During brain development HDACs inhibit BMP2/4 signaling, as this signaling pathway is upregulated, beginning at the transcriptional level, upon inhibition of HDACs by TSA. We observed that TSA inhibition of HDAC activity resulted in an increase of histone H3 and H4 acetylation levels already within one hour of drug application, suggesting that corresponding changes in gene expression could take place very quickly, as has been reported before [62]. In principle, the suppression of BMP signaling could be accomplished at the transcriptional level in a number of ways, including the ligands BMP2/4, their receptors, and genes in the signaling pathway downstream of the BMP ligands, such as the Smad family members. Indeed, within 12 hours of TSA application to cortical or GE-derived precursor cultures, we saw an upregulation in the expression of *Bmp2*, and a down-regulation in the inhibitory cytoplasmic factor *Smad7*. Both of these changes could contribute to an upregulation in the BMP2/4 signaling pathway. To prove this point, extracellular inhibition of BMP2/4 activity by noggin or Alk3-ECD restored both neurogenesis and astrogliogenesis to normal levels in TSA-treated cultures derived from both cortex and GE. Although noggin can inhibit other BMP family members, the binding and inhibition of BMP2 and -4 by Alk3-ECD, the high affinity receptor for these growth factors, identifies this BMP subfamily as responsible for the reported effects. Together, the two complementary transcriptional effects in *Bmp2* and *Smad7* may act in a synergistic fashion to tip the balance from neurogenesis to astrogliogenesis in the GE, and to further reinforce neurogenesis in the cortex. We propose that an important role of HDACs in GE-derived neural progenitor cells is to suppress the responsiveness of the progenitors to astrocyte fate-promoting signals from BMP2 and BMP4. Two lines of evidence suggest that these two factors are produced by the progenitors themselves. *In vitro*, the cultures are homogenous at the level of nestin staining in the minimal time period in which TSA exerts its effects, i.e. in the 24 hour period before bFGF withdrawal (Fig. 4C, D, E). The observed levels of *Bmp2* and *Bmp4* gene expression are therefore probably reflective of production by the nestin-positive progenitors. *In vivo*, direct identification of *Bmp2*- and *Bmp4*-expressing cells by *in situ* hybridization, followed by the labeling of radial glia cells with an antibody recognizing BLBP, clearly identifies radial glia as a significant minority of the cells in the ventricular zone of both GE and cortex that respond to HDAC inhibition by upregulation of *Bmp2* (Fig. 11A, A′).

During mammalian brain development, neural progenitors at first generate primarily neurons and toward the end of gestation switch to the production of astrocytes and oligodendrocytes [1]. How do BMP2/4 affect this switch? In our study, a 24-hour treatment with TSA just before bFGF withdrawal is sufficient to inhibit neurogenesis in GE-derived precursor cultures (Fig. 6A), and up to 4 cell divisions could occur between the cell type that is affected by TSA in this 24-hour period and the generation of neurons or astrocytes 3–4 days later, based upon a calculated cell-cycle length of 21 hours (Maya Shakèd, unpublished data; [75]). The affected cell type could in principle be a bipotential progenitor, but it is more likely to represent a progenitor such as a radial glial cell [76], that gives rise to separate neuroblasts and glioblasts, with BMP2/4 favoring the formation of glioblasts. Indeed, the cell type shown to respond to an upregulation of *Bmp2* transcription in the developing cortex and GE proved to be almost exclusively RC2-positive neural precursor cells, as identified by nuclear staining for phosphorylated Smad1/5/8 (Fig. 10). Interestingly, we saw an increase *in vivo* in the cortex in the RC2-positive population, with a corresponding slight increase in neurogenesis. This would be consistent with a neurogenic role of these precursors at this developmental time point. Indeed, it has been shown that ectopic application of BMP2 or BMP4 to acute telencephalic slices [21] or dissociated cultures [20] from this time period (14.5 d.p.c. in the mouse, E16 in the rat) promotes neurogenesis.

Interestingly, after HDAC inhibition in the GE we saw an opposite effect upon neurogenesis and no such increase in the radial glial population. In contrast to the cortex, radial glia in the GE are poised to start generating astrocytic precursors at 14.5–15.5 d.p.c., the timeframe of our investigation [2]. The advanced developmental state of the radial glia in the GE could explain the relative ease with which HDAC inhibition, leading to an upregulation of BMP2/4 signaling, results in a switch to an astrogliogenic program. In contrast, neocortical radial glia are in the middle of their neurogenic program in the time frame we investigated. In this context, the strong expression of the neurogenic factor *Ngn1* has been shown to actively inhibit astrogliogenesis in the neocortex at this time [77], and indeed, *Ngn1* was seen to be upregulated upon HDAC inhibition in precursor cultures derived from cortical progenitors (Fig. 3J). However, *Ngn1* was also seen to be upregulated by HDAC inhibition in GE-derived precursor cultures (Fig. 3I), but it clearly did not show a neurogenic effect upon the cultures, perhaps explained by the fact that its basal level of transcription is much lower in the GE than in neocortex. Finally, HDACs have catalytic activity upon proteins other than histones, including alpha-tubulin [78], p53, and CBP/p300 [36], so there is also the possibility that HDACs regulate the acetylation of non-histone proteins important for BMP signaling. Clearly, other factors must be at work to explain the difference in response to HDAC inhibition in the cortex and the GE, and these differences will best be investigated by performing an unbiased gene expression analysis using microarray gene chips.

It is unclear why GE precursors do not precociously generate GFAP+ astrocytes *in vivo* after HDAC inhibition, as they certainly do so in precursor cultures derived from the GE (Fig. 5C, D). Premature astrogliogenesis *in vivo* has been seen in several mouse mutants in neurogenic genes [9], [79] and for the DNA-methylating enzyme *Dnmt1* [74]. In the *Dnmt1* mutant, the astrogliogenic transcription factors *Stat1* and *Stat3* are upregulated at the transcriptional level, a change correlating with a demethylation of the *Stat1* gene promoter. Interestingly, we also observed an upregulation of both *Stat1* and *Stat3* in both cortical and GE cultures upon TSA treatment (Fig. 3G, H), without seeing any increase in astrogliogenesis in cortical cultures, which could be explained by the anti-astrogliogenic effect of Ngn1 in cortical precursors [77]. Similarly, it could be that pharmacological modulation of histone acetylation levels is not sufficiently strong to overcome inhibitory signals to astrogliogenesis present in the embryonic GE, and that these inhibitory signals are either absent or reduced in *in vitro* cultures. Indeed, astrogliogenesis was clearly promoted in dissociated cultures prepared from the GE of embryos that had been treated with TSA *in utero* and then treated with either BMP2, LIF, or both factors together (Fig. 9C). However, the nature of these signals remains unclear.

Which HDAC is responsible for the control of neurogenesis in the cortex and GE? Of the five different HDAC inhibitors used in this study, only VPA, which specifically inhibits class I HDACs at the concentrations we have used [58], did not show any affect upon neurogenesis, although it did inhibit the production and maturation of oligodendrocytes, as reported previously [43]. This suggests that class II HDACs may be responsible for controlling neurogenesis in the telencephalon. Of the class II HDACs that we examined, we detected protein expression of HDAC4, -5, -6, and -7 (Fig. 4). The expression of HDAC9 has also been reported at the mRNA level in differentiating neurosphere cultures [80]. As it is possible that these gene products may perform similar functions in the neural progenitors, identification of the individual HDACs involved in neurogenesis may be difficult. In this respect, comparison with myogenesis may prove useful. In the mouse, individual knock-outs of *Hdac4* [48], *Hdac5* [50] and *Hdac7* [52] have not been reported to show a disruption in the skeletal myogenesis program, although all three proteins are clearly important for the *in vitro* differentiation of myoblasts to myotubes [36]. In addition, HDAC5 and HDAC9 have been reported to play functionally redundant roles in heart development *in vivo* [50]. Possible redundancy of HDAC function in neurogenesis from neural stem cells is currently under investigation through sequential and combinatorial knockdown experiments using RNA interference. It will also be important, however, to undertake a closer examination of the various HDAC knockout strains for defects in nervous system development.

Our results stand in agreement to a previous report [81] showing that several HDAC inhibitors, including TSA, VPA, and sodium butyrate, dramatically increase neurogenesis from neurospheres derived from adult rat hippocampus, which develops as an invagination of the dorsal telencephalon at the same time as the cortex. We did not see such a large increase in neurogenesis in embryonic cortex *in vivo*, but we did observe a doubling of neurogenesis *in vitro*. In addition to the difference in species and developmental stage examined in these two studies, these results may reflect the different roles that BMPs play in neurogenesis in various regions of the brain. In the developing cortex, BMP2/4 promote the formation of neurons [21], but in contrast they have been shown to inhibit neurogenesis in neural stem cells derived from the subventricular zone of the embryonic GE [14]. Our data are consistent with an inhibitory role of BMP2/4 upon neurogenesis in the ventral telencephalon. It is of interest to note that ablation of *hdac1* in the embryonic zebrafish also leads to a reduction in neurogenesis, both in the retina [46] and in motor neurons of the spinal cord [47]. In the former case, Hdac1 was found to suppress both Wnt and Notch signaling. We investigated both of these pathways and found that neither of them seem to play a role in the HDAC-mediated promotion of neurogenesis, at least in GE-derived neural progenitor cells. Whether this reflects a vertebrate class difference between the two model organisms or differences in HDAC function between various regions of the nervous system is unclear.

### Conclusions

In this study, HDACs are shown to play an important role in neurogenesis in the developing GE and a modulatory role in neocortical neurogenesis. HDAC control of *Bmp2* and *Smad7* transcription leads to an inhibition of BMP2/4 signaling and thereby a neurogenic program in GE, and in contrast an inhibition of neurogenesis in the cortex. Inhibition of class I and II HDACs with TSA or other HDAC inhibitors causes an increase and a decrease, respectively, in the expression of *Bmp2* and *Smad7*, leading to an elevation in BMP2/4 signaling and a switch to an astrocytic program in the GE and a promotion of neurogenesis in the cortex. Our study suggests that histone acetylation levels play a crucial and similar role in the regulation of the transcription of genes in the BMP signaling pathway in both dorsal and ventral telencephalon, but that this similar modulation of transcription results in opposite biological outcomes. The reason for this difference remains unclear, and therefore future studies will examine this hypothesis in detail, examining global changes as well as specific modifications of core histones at the promoters of genes relevant for BMP2/4 signaling.

## Materials and Methods

All reagents were purchased from Sigma-Aldrich (Taufkirchen, Germany) unless otherwise indicated.

### Mouse lines

All animal experiments were conducted in compliance with the regulations of the state of Baden-Württemberg, Germany. We employed C57BL/6J and CBA/J mice (Charles River, Sulzfeld, Germany) and the strain *Mapt^tm1(GFP)Klt^* [60], which had been backcrossed to wild-type C57BL/6J mice for more than ten generations to generate a congenic line that would avoid possible inconsistencies arising from mixed genetic backgrounds.

### Neurosphere culture

We employed mice of the inbred backgrounds C57BL/6J or CBA/J and the *tauGFP* mouse line. Neurospheres (NS) were prepared from the cortex and the lateral and medial GEs of 15.5–16.0 d.p.c. (Theiler Stage 23/24) embryos, essentially as described [61], with the addition of bFGF at 10 ng/ml (full protocol described in [56]). Embryos were dissected on ice in HBSS (Invitrogen) with 1% HEPES and decapitated. The brain was removed, the hemispheres separated, and the GE removed with fine forceps. To prepare neurosphere cultures from the cortex, the hippocampus and olfactory bulbs were removed from the cerebral hemispheres, and the remaining cortex was dissected out using forceps. GE or cortical cells were mechanically dissociated with a fire-polished (fp) Pasteur pipette and plated out in cell culture flasks with 100,000 cells per milliliter (ml) in NS Medium (F12/DMEM (1∶1) with B27 supplement (Invitrogen), penicillin/streptomycin (100 U/ml, Invitrogen), human EGF (20 ng/ml; Sigma) and human bFGF (10 ng/ml; R&D Systems, Wiesbaden, Germany)). The NS were incubated in suspension at 37°C, 5% CO_2_ for 1 week and fed on the 5^th^ day with an equal volume of NS medium. For differentiation, 7 day-old NS were collected into 50-ml tubes and centrifuged for 3 minutes at 200× g. The NS were mechanically dissociated using a fp Pasteur pipette and plated out with 200,000 cells per well in 12-well plates containing 18-mm coverslips (pre-plated with 200 µg/ml polyornithine) in NS medium without EGF and with 1% fetal calf serum (FCS, Invitrogen), a medium that supports the differentiation of both neurons and astrocytes. After 2.5 days of incubation the medium was changed to NS medium without bFGF and EGF but with 1% FCS. The following pharmacological reagents were added in different experiments: trichostatin A (Sigma, Calbiochem), valproic acid (Sigma, Calbiochem), SAHA (N. Nishino), apicidin (Sigma), and SBHA (Calbiochem). Cells were fixed in 4% PFA for 30 minutes at 4°C at different time points and then processed for cytofluorescence.

### Immunocytofluorescence

Fixed cells were blocked for 1 hour at room temperature (Blocking buffer: 0.5% triton X-100, 1% BSA and 5% NGS in 1× PBS). Blocking buffer did not contain triton when O4 antibody was used. Abs were diluted in blocking buffer, applied overnight, and washed 4 times in PBS, followed by incubation with appropriate secondary antibodies, washing, and mounting for microscopy, as described below for immunohistofluorescence. Primary Abs were diluted as follows: anti-nestin (Rat-401 clone; BD Pharmingen) 1∶1000, anti-β tubulin III (TuJ1 clone; Covance and R&D Systems) 1∶1500, anti-GFAP (GA5 clone; Sigma and rabbit polyclonal Z0334; DAKO Cytomation, Hamburg, Germany) 1∶500, O4 (kind gift of Prof. J. Trotter, Mannheim, Germany) 1∶100, anti-GFP (kind gift of U. Müller) 1∶1000, and anti-phosphorylated Smad1/5/8 (rabbit 9511; Cell Signaling) 1∶1000. Proliferation was measured using BrdU labeling (10 µM) for 24 hours followed by immunohistofluorescence detection using an anti-BrdU Ab (clone BU33; Sigma) 1∶1000. Apoptosis was measured using ApopTag Red *in situ* apoptosis detection kit (Chemicon). Secondary antibodies were used as described below for immunohistofluorescence.

### SAHA synthesis

SAHA was synthesized as described [82].

### Recombinant proteins

The recombinant proteins human BMP2 (rhBMP2), human Noggin (rhNoggin), and the extracellular domain of the human BMP receptor Alk3 (BMPR1A, residues 24–152) (rhAlk3-ECD) were expressed as maltose-binding protein (MBP) fusion proteins in *E. coli*. The coding sequences were fused in frame to MBP into a malE fusion vector derived from pMALc2X (New England Biolabs, Frankfurt, Germany) in which the factor Xa site is replaced with a 6x-His tag and a thrombin-cleavage site (LVPRGS) [65]. rhBMP2 was expressed after IPTG induction of transformed *E. coli* cultured in a glucose/mineral salt medium. MBP-BMP2 fusion proteins were purified by amylose affinity chromatography, followed by *in vitro* dimerization and final purification of dimeric rhBMP2 after thrombin cleavage as described [65], [83]. Activity of rhBMP2 was tested by its ability to induce alkaline phosphatase in the myoblast line C2C12, which is associated with cellular differentiation [84]. rhNoggin and rhAlk3-ECD were prepared similarly, with the exception that rhAlk3-ECD did not require an *in vitro* dimerization step. The functionality of rhAlk3-ECD was demonstrated by surface plasmon resonance (BIAcore) analysis. Using induction of alkaline phosphatase in C2C12 cells in response to rhBMP2 as an activity readout, the activity of rhBMP2 could be suppressed by rhNoggin as well as rhAlk3-ECD at appropriate concentrations in tissue culture assays (data available upon request). Recombinant murine Dkk1 and sFRP2 were purchased from R&D Systems.

### Western blots

Protein lysates from NS cultures were made using RIPA buffer and proteinase inhibitors (Roche, Mannheim, Germany). Protein concentrations were measured using the Bradford assay (Bio-Rad Laboratories, Munich, Germany). 25 µg lysates were electrophoresed in 10–15% acrylamide gels, and transfer was done using the Bio-Rad Mini-PROTEAN 3 System, followed by Western blots as described [60] using the following primary antibodies: anti-HDAC antibodies: HDAC1, HDAC3, HDAC4, HDAC5, HDAC6, and HDAC7 (Cell Signaling Technology) 1∶1000; HDAC2 (ZYMED Laboratories, San Francisco, CA) 1∶2000; anti-cleaved Notch1 (Val1744, rabbit polyclonal; Cell Signaling) 1∶500, anti-acetylated histone H3 (Lys9/18; Upstate) 1∶1000, anti-acetylated histone H4 (rabbit antiserum 06-866; Upstate) 1∶500, anti-α tubulin (Clone DM1A, Sigma) 1∶5000, and anti-β actin (clone AC-15, Sigma) 1∶1000. Secondary anti-rabbit and anti-mouse antibodies were conjugated to horseradish peroxidase (dianova, Hamburg, Germany) 1∶10,000. Blots were developed using either the ECL Advance or Plus Western Blotting Detection Kit (GE Healthcare, Munich, Germany) and exposed to Hyperfilm ECL (GE Healthcare).

### RT-PCR Analysis

All reagents from Invitrogen except where noted. RNA was isolated from neural stem cell cultures that had been plated out upon 10-cm polyornithine-coated tissue-culture dishes and cultured for 2.5 days. TRIzol extraction of RNA, followed by DNAse I (Fermentas, St. Leon-Rot, Germany) treatment and conversion of 1 µg of RNA using oligo(dT)_12–18_ and SuperScript II RNase H^−^ reverse transcriptase into cDNA was performed according to manufacturer specifications. The following intron-spanning primers (Thermo Electron, Ulm, Germany) were used for 30–45-cycle PCR using 60°C for annealing: BMP2: 5′-ctcagcgaatttgagttgagg, 5′-gttttcccactcatctctgg; BMP4: 5′-aaagtcgccgagattcagg, 5′-gaggaaacgaaaagcagagc; and GAPDH: 5′-accacagtccatgccatcac, 5′-tccaccaccctgttgctgta.

### RNA extraction and quantitative real time RT-PCR

Total RNA was isolated from neurosphere cultures that had been plated out upon polyornithine and cultured for 2.0 days. RNA was extracted using the RNeasy Mini Kit (74104 Qiagen) according to the manufacturer's instructions. 1–5 µg of RNA was transcribed into cDNA using oligo(dT)_12–18_ (0,5 µg/µl, Invitrogen) or random hexamers (50 mM, Applied Biosystems, Darmstadt, Germany) and SuperScript II RNase H^−^ reverse transcriptase (Invitrogen). Quantitative real time PCR reactions were performed using the ABI Prism 7000 Detection System (Applied Biosystems) using TaqMan Gene Expression Assays (Applied Biosystems) with 1 µl cDNA (20 µl RTase reactions using 1–5 µg whole RNA input). The following TaqMan Assays were used: *Bmpr1a* (Mm00477650_m1), *Bmpr1b* (Mm00432117_m1), *Bmpr2* (Mm00432117_m1), *Bmp2* (Mm01340178_m1), *Bmp4* (Mm00432087_m1), *Stat1* (Mm00434761_m1), *Stat3* (Mm00456961_m1), *Ngn1* (Mm00440466_s1), *Ngn2* (Mm00437603_g1) and *Smad7* (Mm00484741_m1). The standard quantification protocol was applied with the following cycles: 2 min at 50.0° C, 10 min at 95.0° C, followed by 45 cycles: 15 seconds at 95.0° C and 1 min at 60.0°C. Each individual reaction was performed in triplicate. GAPDH primers (Mm99999915_g1) were used to normalize results presented in the paper. HPRT primers (Mm00446968_m1) were used as controls in independent and simultaneous experiments to exclude the possibility that GAPDH expression was regulated by HDAC inhibition. Although individual experiments produced differing relative expression values using the two control genes, the directional trends with either control were always the same. Furthermore, statistical analysis performed using either GAPDH or HPRT as a control did not show any significant differences between the two data sets.

Statistical analysis was performed as follows: Relative expression (RE) levels were calculated with the function (RE = 2^−ΔΔCt^), where ΔΔCt is the normalized difference in threshold cycle (Ct) number between a treatment of cultures with vehicle (0.008% ethanol) or with TSA, calculated from the mean Ct value of triplicate replicates of any given condition. The mean of RE reported in Fig. 7 was calculated from the individual RE values from 4–8 independent experiments, and the standard error of the mean (SEM) was calculated from the standard deviation of the 4–8 RE values. Statistical significance was evaluated by applying the Student's T-test to the 4–8 RE values, comparing vehicle to TSA treatment. Application of Student's T-test to the original ΔΔCt values produced comparable p values (data available upon request).

### Data collection and analysis

Fluorescence photographs were taken with an Olympus BX61WI microscope with an F-view II 12-Bit monochromatic CCD camera (Soft Imaging System, Muenster, Germany) and analyzed using the analySIS program (Soft Imaging System). Images in Fig. 7, 8, and 10 were produced using a Nikon C1Si spectral confocal laser scanning system and a Nikon TE2000-E inverted microscope.

In all cases where data is expressed as a percentage (e.g. percentage neurons), statistical significance was assayed for each experiment using a chi-squared analysis. The mean was calculated of the percentage values from multiple, independent experiments under a given condition and then compared with the mean for other conditions (e.g. TSA treatment) using a Mann-Whitney U test upon the means derived from individual experiments. Similar analyses using ANOVA and the Student's t-test yielded only lower p values. Non-categorical data were evaluated using the Student's t-test, as described above for RT-PCR.

### TSA injections

13.25 d.p.c. pregnant *tauGFP* [85] females were injected intraperitoneally every 12 hours with either vehicle (8% ethanol in 1× PBS) or TSA (Sigma, Calbiochem, Schwalbach, Germany; 0.5 mg/kg body weight) dissolved in vehicle (100 µg/ml). Females were sacrificed at 15.75 d.p.c., after a total of five injections. Embryos were removed and decapitated. Embryonic heads were either fixed in 4% PFA or dissected (GE and cortex) for FACS analysis / sorting and for acute dissociated cultures. For this latter procedure, cells were lightly trypsinized, mechanically dissociated, and plated out for 2–3 hours in neurosphere culture medium onto 18-mm glass coverslips coated with poly-ornithine, fixed with 4% PFA, and then processed for immunocytofluorescence as described below.

### FACS analysis

Whole brains were removed from the head, cerebral hemispheres separated, and meninges removed. Medial and lateral GE were carefully removed with fine forceps and collected into Eppendorf tubes. Hippocampus and olfactory bulbs were removed from the remaining hemispheres, and the remaining cortex was dissected out using forceps. Cells were lightly trypsinized and then mechanically dissociated to a single-cell suspension with a fp Pasteur pipette. FACS analysis was performed on a FACSCalibur device (BD Biosciences, San Jose, CA). Just before the analysis, propidium iodide (PI; 250 ng/ml) was added to allow exclusion of dead cells by use of an appropriate gate. A proper compensation for the GFP-based fluorescent signal was first determined using cells not labeled with PI. 50,000 events were counted for each condition, with a sort rate of <1,000 events per second. Non-transgenic embryos were used as a negative control to estimate background (less then 1%).

### FACS sorting

FACS sorting was performed using a FACSort device (BD Biosciences) in the single cell mode (75–100 cells per second). Identical controls as above were used to determine background fluorescence of GFP-negative cells and for compensation for the GFP-based signal. To determine the appropriate gating for FACS analysis, cells were plated for 2–3 hours onto 18-mm glass coverslips coated with poly-ornithine (200 µg/ml) immediately after sorting, followed by staining for GFP and β-tubulin III, as described above.

### Acute dissociated cell culture

13.25 d.p.c. pregnant *tauGFP* females were injected intraperitoneally every 12 hours with either vehicle or TSA, as described above. Females were sacrificed at 15.75 d.p.c., after a total of five injections. Embryos were removed and decapitated. GE and cortex were dissected and plated out at a density of 300,000 cells per well in 12-well plates containing 18-mm coverslips (pre-plated with 200 µg/ml polyornithine) in NS medium without EGF and with 1% fetal calf serum (differentiation medium). BMP2 (10 ng/ml), LIF (25 ng/ml), or both factors were added for 12 hours after plating out and removed at 2.5 DIV, at the same time that bFGF was withdrawn. Cells were cultured in differentiation medium without bFGF, fixed at 7 days in 4% PFA, and stained with an anti-GFAP antibody and DAPI.

### Immunohistofluorescence

Embryos and sections were prepared as described [86]. Heads were fixed in 4% PFA at 4°C overnight, transferred to 30% sucrose in 1× PBS overnight, and then mounted on dry ice in tissue freezing medium (Jung, Leica, Nussloch, Germany). 12 µm coronal sections were cut on a cryostat (Leica CM3050 S) and then blocked for several hours at room temperature (Blocking buffer: 0.5% triton X-100, 1% bovine serum albumin (BSA) and 5% native goat serum (NGS) in 1× PBS. Primary antibodies (Ab) were diluted in blocking buffer as follows and incubated overnight at 4°C: anti-acetyl-histone H3 (Lys9/18, rabbit antiserum 07-593; Upstate USA, Charlottesville, VA) 1∶400, anti-β tubulin III (TuJ1 clone; Covance, Berkeley, CA) 1∶500, anti-GABA (rabbit A2052; Sigma) 1∶400, anti-phospho-histone H3 (Ser10, rabbit 06-570; Upstate) 1∶200, anti-cleaved caspase-3 (Asp175, 5A1, rabbit mAb; Cell Signaling Technology, Frankfurt, Germany) 1∶200, RC2 (Developmental Studies Hybridoma Bank, Iowa City, Iowa) 1∶10, anti-nestin (Rat 401 clone; BD Pharmingen, Heidelberg, Germany) 1∶500, anti-Pax6 (rabbit AB5409; Chemicon, Chandler's Ford, UK) 1∶500, anti-S-100 (β subunit, clone SH-B1; Sigma) 1∶500, anti-BLBP (rabbit AB9558, Chemicon) 1∶2000, and anti-phospho-Smad1/5/8 (rabbit 9511; Cell Signaling) 1∶500. After washing slides 4 times with 1× PBS, the secondary Abs (goat anti-rabbit AlexaFluor 488 or 555 and goat anti-mouse AlexaFluor 488 or 555; Invitrogen, Karlsruhe, Germany) were applied for 1 hour at room temperature, followed by DAPI (2.5 µg/ml) staining, washed 3 times in PBS, and mounted for microscopy (Aqua PolyMount, Polysciences Europe, Eppelheim, Germany).

### In situ hybridization

*In situ* hybridization on paraffin sections was performed as described [87], with the following buffer used for the final washing and developing steps: 100 mM NaCl, 50 mM MgCl_2_, 100 mM Tris-HCl, pH 9.5, 0.1% Tween-20. Antisense probes for *Bmp2* and *Bmp4* were prepared from plasmids containing portions of the murine cDNAs (*Bmp2*: bp 336–998 of the NM007553 reference cDNA, *Bmp4*: bp 430–1316 of the NM007554 reference cDNA), subcloned into the pGEM-T vector (kind gift of K. Huber). *In situ* hybridization was followed by immunohistofluorescence with the anti-BLBP antibody, as described above.

## Supporting Information

Figure S1 A multitude of class I and II HDAC genes are expressed in differentiating neural progenitor cultures derived from embryonic GE. (A) Neurosphere cultures derived from 15.5 d.p.c. mouse GE were dissociated, cultured on polyornithine, and the mitogen bFGF was removed from the cultures at 2.5 days in vitro (DIV). Cultures were collected at day 0 and after 1, 2.5, 3.5, and 7 DIV. Protein lysates were electrophoresed in 10–15% SDS-PAGE gels and transferred to PVDF membranes. HDAC1, -2, -3, -4, -5, -6 and -7 were detected using specific polyclonal antibodies. Loading levels were confirmed by reprobing each blot with an antibody recognizing α-tubulin (below each respective anti-HDAC panel). (B) Cultures were stained at 1 DIV with an antibody recognizing nestin (red), expressed by neural precursors. DAPI staining of the nucleus is shown in green. Scale bar = 25 µm. (C) The percentage of nestin+ cells present at progressive DIV was calculated using a nuclear DAPI stain to evaluate total cell number. n = 2. (D) TSA treatment did not affect the initial number of nestin+ precursors, evaluated at 1 DIV after 24 hours exposure to TSA.
(9.79 MB TIF)

Figure S2 Various HDAC inhibitors can inhibit neurogenesis and promote astrogliogenesis in differentiating neural progenitor cultures derived from embryonic GE. Dissociated neurospheres were plated onto coverslips and treated with the indicated inhibitors of class I and II HDACs for 1 week of in vitro differentiation, then stained with antibodies against β-tubulin III to detect neurons (TuJ1) (A) or against GFAP to detect astrocytes (B). The percentage of cells detected with each antibody is indicated. Mean values +/− SEM (n = 3). * = p<0.05, ** = p<0.01, *** = p<0.001, Mann-Whitney U test.
(3.36 MB TIF)

Figure S3 Inhibition of class I and II HDACs by TSA in differentiating neural progenitor cultures derived from embryonic GE results in an increase in nestin-positive precursors and a decrease in oligodendrocytes. Dissociated neurospheres were plated onto coverslips and treated with 10 nM TSA for 1 week of in vitro differentiation, then stained with antibodies (red) against nestin to detect dividing neural precurors (A) or with the O4 antibody (red) to detect oligodendrocytes (B). DAPI (green) was used to stain cell nuclei. Scale bar = 100 µm. (C, D) The percentage of cells detected with each antibody is indicated. Mean values +/− SEM (n = 3). * = p<0.05, *** = p<0.001, Mann-Whitney U test.
(5.78 MB TIF)

Figure S4 Notch and canonical Wnt signaling pathways are not involved in inhibition of neurogenesis by TSA in differentiating neural progenitor cultures derived from embryonic GE. (A) Neurosphere cultures derived from 15.5 d.p.c. GE were dissociated, cultured on polyornithine, and collected at day 0 and after 1.5, 2.5, 3.5, and 7 days in vitro (DIV). The mitogen bFGF was removed from the cultures at 2.5 DIV. Protein lysates were electrophoresed in 10–15% SDS-PAGE gels and transferred to PVDF membranes. The cleaved intracellular domain (ICD) of Notch1 was detected using a polyclonal antibody, and no changes were seen in the relative expression pattern after treatment with 10 nM TSA. Loading levels were confirmed by reprobing each blot with an antibody recognizing α-tubulin (below each respective anti-Notch1 panel). (B) Inhibition of Wnt signaling does not rescue neurogenesis in TSA-treated cultures. Dissociated neurospheres were plated onto coverslips, and at 1.5 DIV cultures were treated with the Wnt signaling inhibitors Dickkopf1 (Dkk1) or secreted Frizzled-related protein 2 (sFRP2), with or without 10 nM TSA. All inhibitors and bFGF were withdrawn 24 hours later. Cells were then cultured for an additional 4.5 days and analyzed by immunofluorescence, staining with the TuJ1 antibody to detect neurons. Mean values +/− SEM (n = 2). * = p<0.05, *** = p<0.001, Mann-Whitney U test.
(0.79 MB TIF)
