# Supplementary figures and images for: Histone Deacetylases Control Neurogenesis in Embryonic Brain by Inhibition of BMP2/4 Signaling
Source: PLoS One. 2008 Jul 16;3(7):e2668. doi: 10.1371/journal.pone.0002668 (PMC2441862; doi:10.1371/journal.pone.0002668)

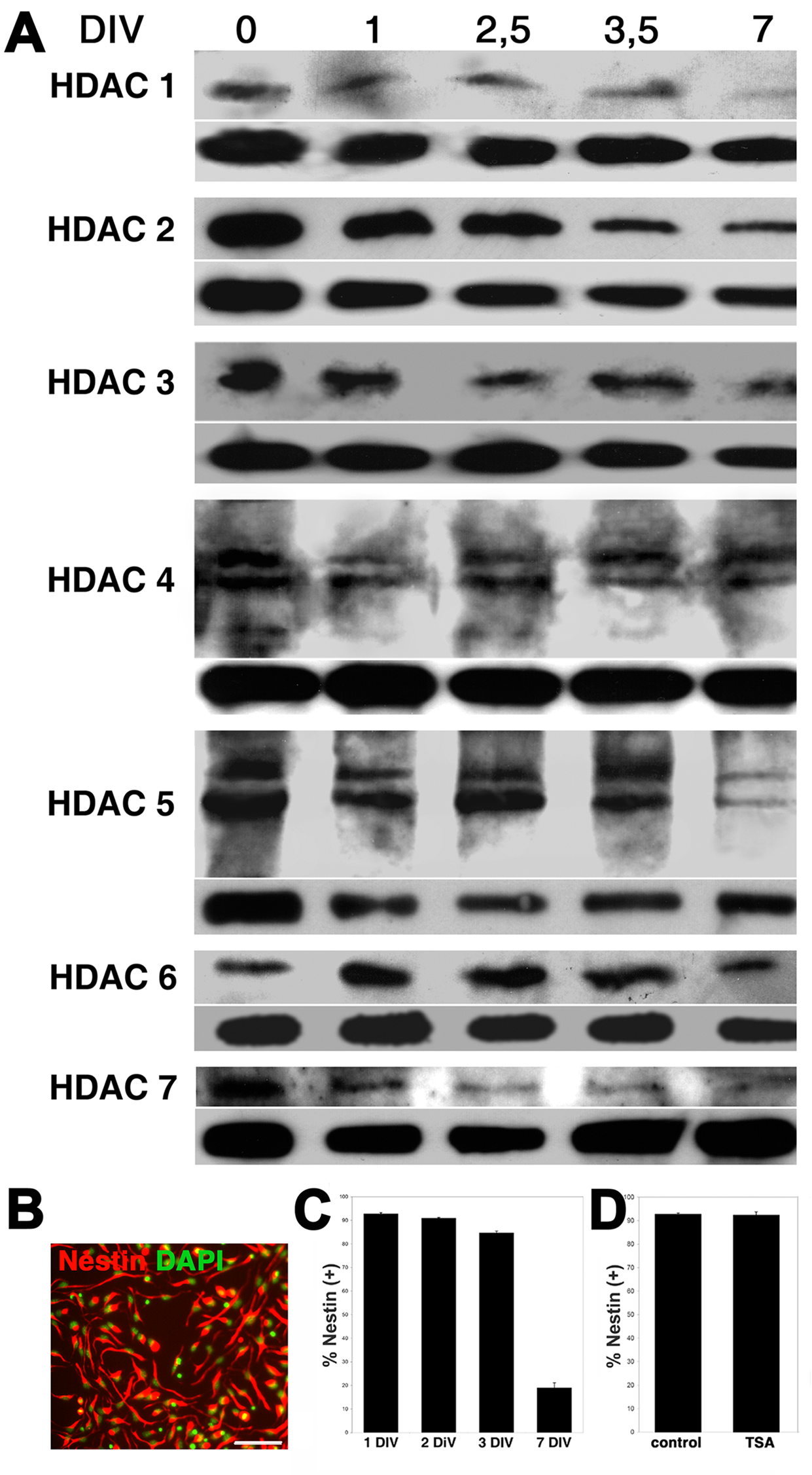

Supplement: Figure S1 — A multitude of class I and II HDAC genes are expressed in differentiating neural progenitor cultures derived from embryonic GE. (A) Neurosphere cultures derived from 15.5 d.p.c. mouse GE were dissociated, cultured on polyornithine, and the mitogen bFGF was removed from the cultures at 2.5 days in vitro (DIV). Cultures were collected at day 0 and after 1, 2.5, 3.5, and 7 DIV. Protein lysates were electrophoresed in 10–15% SDS-PAGE gels and transferred to PVDF membranes. HDAC1, -2, -3, -4, -5, -6 and -7 were detected using specific polyclonal antibodies. Loading levels were confirmed by reprobing each blot with an antibody recognizing α-tubulin (below each respective anti-HDAC panel). (B) Cultures were stained at 1 DIV with an antibody recognizing nestin (red), expressed by neural precursors. DAPI staining of the nucleus is shown in green. Scale bar = 25 µm. (C) The percentage of nestin+ cells present at progressive DIV was calculated using a nuclear DAPI stain to evaluate total cell number. n = 2. (D) TSA treatment did not affect the initial number of nestin+ precursors, evaluated at 1 DIV after 24 hours exposure to TSA. (9.79 MB TIF) [file pone.0002668.s001.tif]

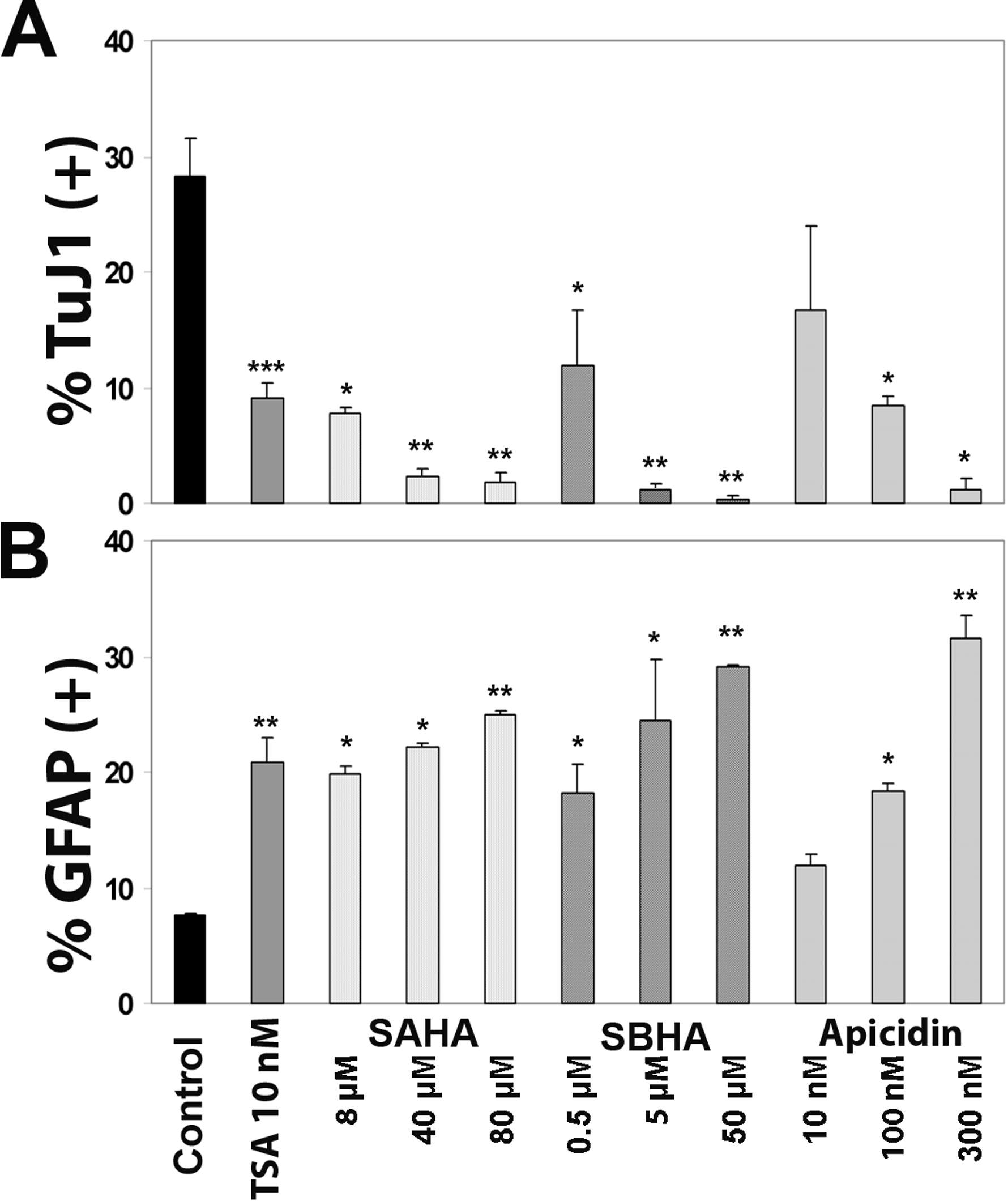

Supplement: Figure S2 — Various HDAC inhibitors can inhibit neurogenesis and promote astrogliogenesis in differentiating neural progenitor cultures derived from embryonic GE. Dissociated neurospheres were plated onto coverslips and treated with the indicated inhibitors of class I and II HDACs for 1 week of in vitro differentiation, then stained with antibodies against β-tubulin III to detect neurons (TuJ1) (A) or against GFAP to detect astrocytes (B). The percentage of cells detected with each antibody is indicated. Mean values +/− SEM (n = 3). * = p<0.05, ** = p<0.01, *** = p<0.001, Mann-Whitney U test. (3.36 MB TIF) [file pone.0002668.s002.tif]

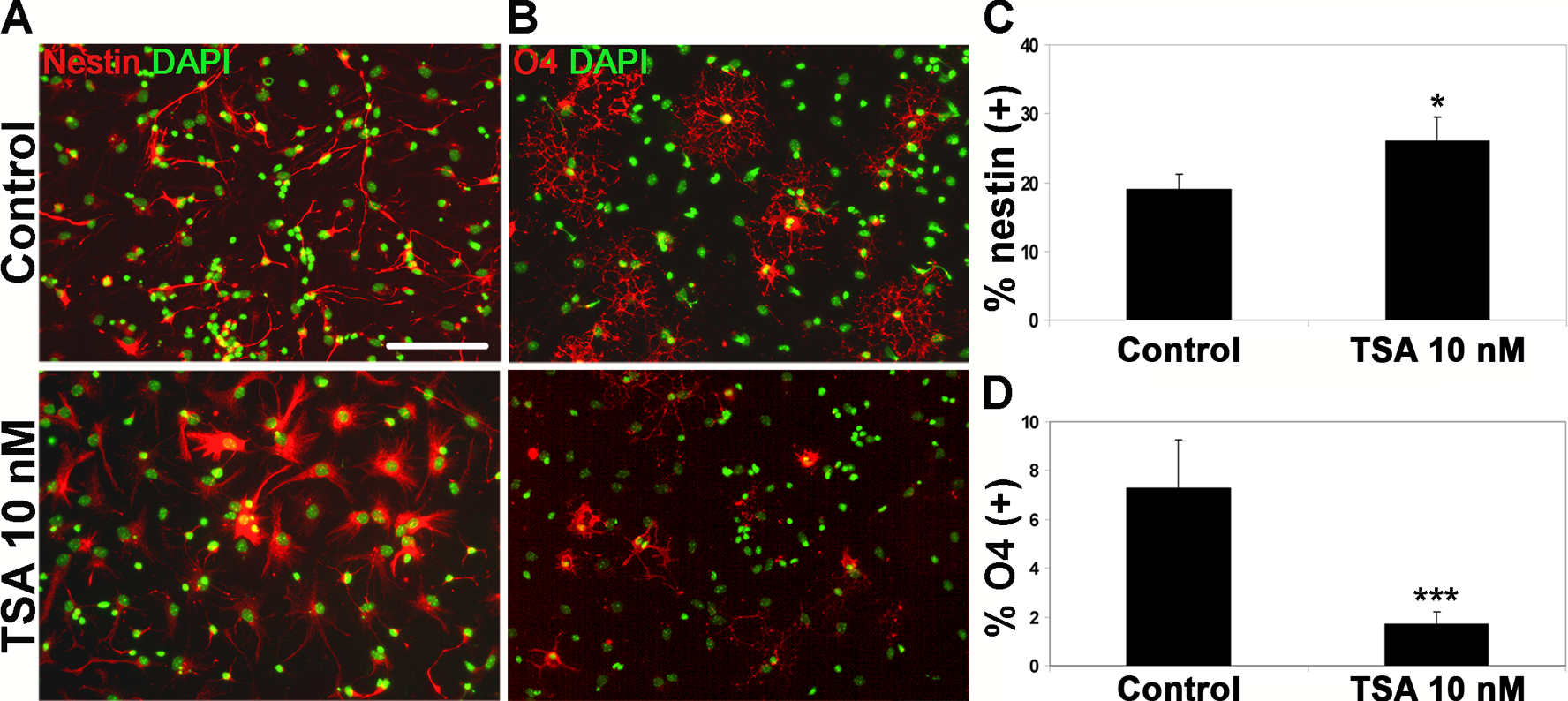

Supplement: Figure S3 — Inhibition of class I and II HDACs by TSA in differentiating neural progenitor cultures derived from embryonic GE results in an increase in nestin-positive precursors and a decrease in oligodendrocytes. Dissociated neurospheres were plated onto coverslips and treated with 10 nM TSA for 1 week of in vitro differentiation, then stained with antibodies (red) against nestin to detect dividing neural precurors (A) or with the O4 antibody (red) to detect oligodendrocytes (B). DAPI (green) was used to stain cell nuclei. Scale bar = 100 µm. (C, D) The percentage of cells detected with each antibody is indicated. Mean values +/− SEM (n = 3). * = p<0.05, *** = p<0.001, Mann-Whitney U test. (5.78 MB TIF) [file pone.0002668.s003.tif]

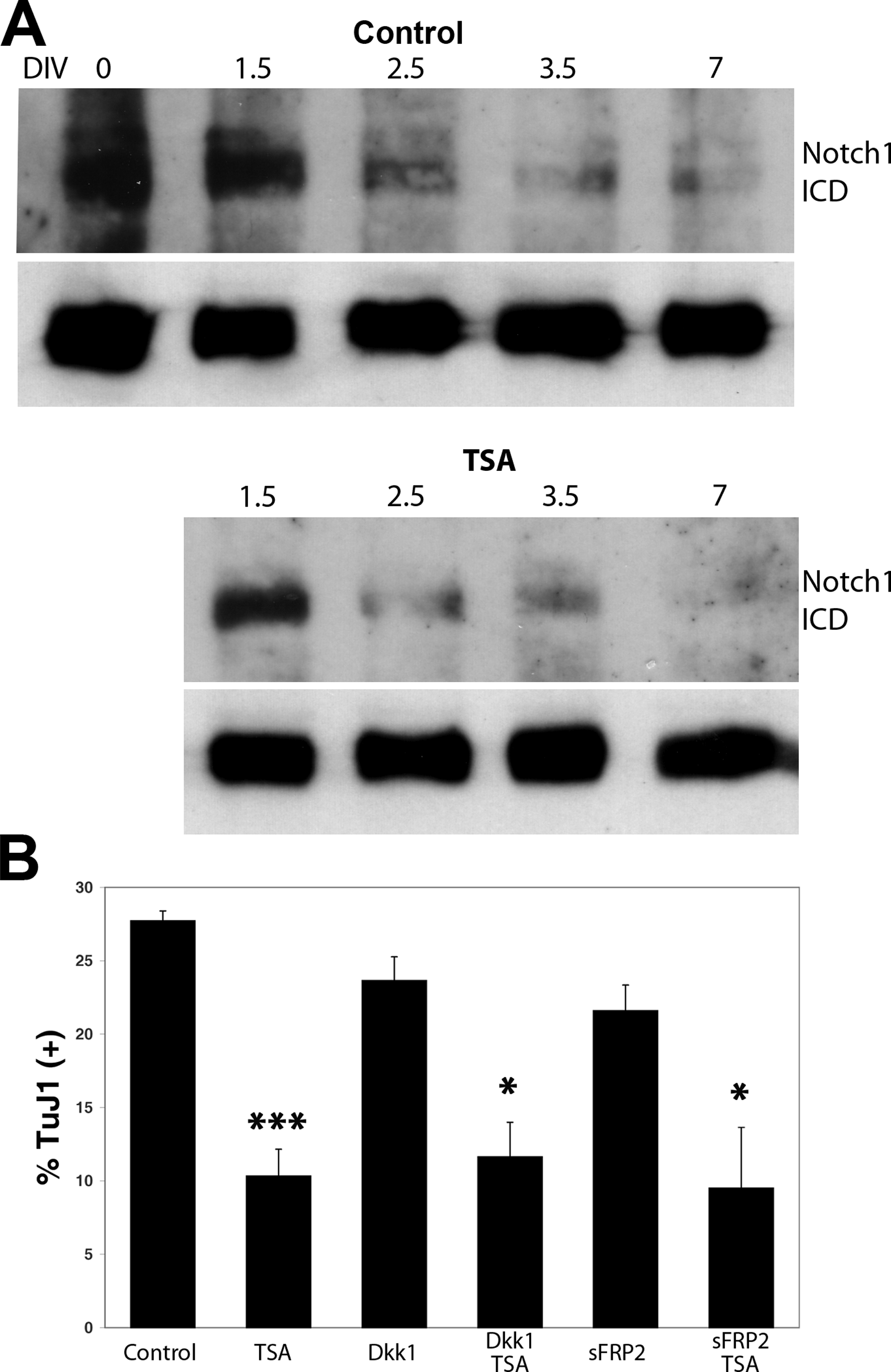

Supplement: Figure S4 — Notch and canonical Wnt signaling pathways are not involved in inhibition of neurogenesis by TSA in differentiating neural progenitor cultures derived from embryonic GE. (A) Neurosphere cultures derived from 15.5 d.p.c. GE were dissociated, cultured on polyornithine, and collected at day 0 and after 1.5, 2.5, 3.5, and 7 days in vitro (DIV). The mitogen bFGF was removed from the cultures at 2.5 DIV. Protein lysates were electrophoresed in 10–15% SDS-PAGE gels and transferred to PVDF membranes. The cleaved intracellular domain (ICD) of Notch1 was detected using a polyclonal antibody, and no changes were seen in the relative expression pattern after treatment with 10 nM TSA. Loading levels were confirmed by reprobing each blot with an antibody recognizing α-tubulin (below each respective anti-Notch1 panel). (B) Inhibition of Wnt signaling does not rescue neurogenesis in TSA-treated cultures. Dissociated neurospheres were plated onto coverslips, and at 1.5 DIV cultures were treated with the Wnt signaling inhibitors Dickkopf1 (Dkk1) or secreted Frizzled-related protein 2 (sFRP2), with or without 10 nM TSA. All inhibitors and bFGF were withdrawn 24 hours later. Cells were then cultured for an additional 4.5 days and analyzed by immunofluorescence, staining with the TuJ1 antibody to detect neurons. Mean values +/− SEM (n = 2). * = p<0.05, *** = p<0.001, Mann-Whitney U test. (0.79 MB TIF) [file pone.0002668.s004.tif]
